# Evaluating red tide effects on the West Florida Shelf using a spatiotemporal ecosystem modeling framework

**DOI:** 10.1038/s41598-023-29327-z

**Published:** 2023-02-13

**Authors:** Daniel Vilas, Joe Buszowski, Skyler Sagarese, Jeroen Steenbeek, Zach Siders, David Chagaris

**Affiliations:** 1 Fisheries and Aquatic Sciences Program, School of Forest Resources and Conservation, University of Florida, Gainesville, FL 32611 USA; 2 Nature Coast Biological Station, Institute of Food and Agricultural Sciences, University of Florida, Cedar Key, FL 32625 USA; 3 Present Address: School of Aquatic and Fishery Sciences, University of Washington, Box 355020, Seattle, WA 98195 USA; 4 Present Address: Resource Assessment and Conservation Engineering Division, Alaska Fisheries Science Center, National Marine Fisheries Service, NOAA, Seattle, WA 98115 USA; 5 Ecopath International Initiative, Barcelona, Spain; 6 NOAA Fisheries Service – Southeast Fisheries Science Center, Miami, FL 33149 USA

**Keywords:** Environmental impact, Marine biology, Community ecology, Ecological modelling, Ecosystem ecology, Ecosystem services, Population dynamics

## Abstract

The West Florida Shelf (WFS), located in the eastern Gulf of Mexico, fosters high species richness and supports highly valuable fisheries. However, red tide events occur regularly that can impact fisheries resources as well as ecosystem state, functioning, and derived services. Therefore, it is important to evaluate and quantify the spatiotemporal impacts of red tides to improve population assessments, mitigate potential negative effects through management, and better understand disturbances to support an ecosystem-based management framework. To model red tide effects on the marine community, we used Ecospace, the spatiotemporal module of the ecosystem modeling framework Ecopath with Ecosim. The inclusion of both lethal and sublethal response functions to red tide and a comprehensive calibration procedure allowed to systematically evaluate red tide effects and increased the robustness of the model and management applicability. Our results suggest severe red tide impacts have occurred on the WFS at the ecosystem, community, and population levels in terms of biomass, catch, and productivity. Sublethal and indirect food-web effects of red tide triggered compensatory responses such as avoidance behavior and release from predation and/or competition.. This study represents a step forward to operationalize spatiotemporal ecosystem models for management purposes that may increase the ability of fisheries managers to respond more effectively and be more proactive to episodic mortality events, such as those caused by red tides.

## Introduction

Globally, harmful algal blooms (HABs) are considered a growing concern because of their increasing frequency and impacts^1^. HABs refer to episodic proliferations of specific micro or macroalgae species that cause toxicity in the waters or lead to hypoxia. Although HABs are often localized and short in duration, severe blooms can inflict damage on the marine environment, have adverse effects on human health, and impact the fishing and tourism economies^2^. HABs can affect the quality of the environment and thus the ecosystem and marine species, including endangered and protected species^3,4^. HABs may disturb marine communities by inducing shifts in the spatiotemporal distribution of fish species, causing massive mortality and impacting the whole ecosystem^5–7^. In the US, the estimated annual economic impact due to HABs on commercially harvested fish and shellfish in 2018 and aquaculture production in 2017 were $5.6 billion and $1.5 billion, respectively^8^.

On the West Florida Shelf (WFS) in the Gulf of Mexico, red tides are a type of HAB that are caused by the toxic dinoflagellate *Karenia brevis*^9,10^. *K. brevis* is often present in background concentrations along the WFS, but in some years severe blooms can form, usually along the southwest coast of Florida and late in the summer. Red tides occur in the Gulf of Mexico almost annually and were first recorded in 1874^11^. The exact cause of severe red tide blooms remains unknown but several anthropogenic and natural processes have been suggested as the causative factor. Local winds and deep-ocean forcing effects influence the coastal ocean circulation that can alter the nutrient state of the WFS and influence the occurrence of *K. brevis*^12–14^. Iron deposition from Saharan dust was suggested to be another possible cause of red tide blooms in the Gulf of Mexico^15^. Nutrient enrichment from human activities has also been identified as a potential driver of red tide blooms, especially in areas with high agricultural production^16,17^. Red tides have attracted major attention of policymakers, the scientific community, and society due to the complexity of the process and negative effects on the environment, human health, and coastal economies^18,19^.

Red tides present a great challenge for Gulf of Mexico fisheries assessment and management because of the sudden and localized nature of these events and the difficulties in quantifying fish mortality rates and impacts on stock productivity. Red tides have been incorporated into previous stock assessment models^20,21^ of red grouper (*Epinephelus morio*) and gag grouper (*Mycteroperca microlepis*) as a discard only pseudo-fishing fleet with selectivity assumed to be constant across size and age. During years in which severe red tide events occurred, a mortality rate for the red tide fleet (natural mortality due to red tide events) was estimated to improve the models’ fit to indices of abundance. For gag grouper, the mortality due to a severe red tide event in 2005 was estimated between 0.35 and 0.99 year^−1^ in previous stock assessments^20,22–24^, roughly two to five times higher than baseline natural mortality rates. Similarly, red tides were included in the last stock assessment of red grouper and red tide was estimated to reduce total population biomass by approximately 30% in 2005 and by 21% in 2014^21^. Thus, recent stock assessments have estimated severe, although highly uncertain, effects of red tides on grouper stocks. The uncertainty associated with estimating red tide mortality within a stock assessment framework calls for spatially explicit, independent approaches to quantify red tide mortality that represent the localized and short-lived nature of red tide blooms, as well as the complex effects they have on species and ecosystems.

Ecosystem models offer the ability to integrate, connect, and describe multiple ecosystem components and processes under a single modeling framework^25,26^. Recently, several ecosystem models (e.g. Ecopath with Ecosim) have been developed to investigate potential effects of red tides on the food-web using the non-spatial Ecosim model and including red tides as a pseudo-fishing fleet. For instance, Gray DiLeone and Ainsworth^27^ concluded that red tides can lead to changes in community structure and cascading effects through reduced predator abundances, resulting in predation mortality release for piscivores, planktivores, and detritivores, and triggering an increase in the biomass of these guilds. However, Perryman et al.^28^ indicated that elevated red tide mortality does not impact the ecosystem structure, but it could have meaningful negative effects on natural mortality rates and biomass dynamics of marine species. Although these studies represented a step forward to understand ecosystem impacts due to red tides, their major limitation was the lack of a spatiotemporal framework in which red tides were explicitly considered along with species distribution patterns to comprehensively evaluate their impacts.

Moving to a spatiotemporal ecosystem modeling framework requires consideration of the severity and duration of red tide blooms, their overlap with species of concern, lethal effects, sublethal effects (feeding, growth, and movement) that affect distributions and reduce fitness^29^, and food web impacts. Lethal and sublethal effects of red tides have been identified to impact marine species^3^ which, by altering prey-predator interactions, may lead to cascading effects^30^, thereby affecting the entire ecosystem. Thus, behavior, fitness, and mortality must be spatiotemporally considered to capture red tides effects which can be substantial on certain species depending on the spatial overlap. Spatiotemporal ecosystem models have been successfully implemented to investigate other spatial perturbations and management questions such as placement of marine protected areas^31^, hypoxic events^32^, habitat restoration^33^, eutrophication^34^, invasive species^35^, fishing and climate change^36^ and multiple combined stressors^37^. Besides addressing ecological questions, spatiotemporal ecosystem modeling tools offer the possibility to quantify impacts from episodic events such as red tides in a way that can assist fisheries stock assessments and natural resource management.

This study provides a comprehensive assessment of red tides impacts on the WFS at multiple levels of a marine system. The main objectives are to (i) incorporate spatiotemporal stressors into ecosystem models and estimate and assess their impacts at the ecosystem, community, and population levels by focusing on gag grouper, (ii) tease apart direct effects of red tide from indirect effects arising through perturbations on the food web, and (iii) improve the parametrization process and the robustness of spatiotemporal ecosystem models contributing to ecosystem-based fisheries management. To achieve these objectives, we updated an existing WFS spatiotemporal ecosystem model developed using Ecopath with Ecosim (EwE)^38,39^ to include red tides and their effects on key fishery species and the ecosystem. This included defining species environmental response functions, performing sensitivity analyses, validating and calibrating model predictions, and evaluating uncertainty surrounding red tide effects. We point out that our objective was not to model red tide dynamics or forecast red tide blooms. Rather, our goal was to integrate empirical data on current and past red tides into a fisheries ecosystem model. This study represents the first spatially explicit, comprehensive model developed to represent and estimate red tide impacts on the WFS ecosystem, which may be critical for future management in terms of adaptation, mitigation, and anticipation to red tides considering that their impacts are expected to increase over time^8^.

## Methods

### The West Florida Shelf ecosystem model

**Figure 1:**
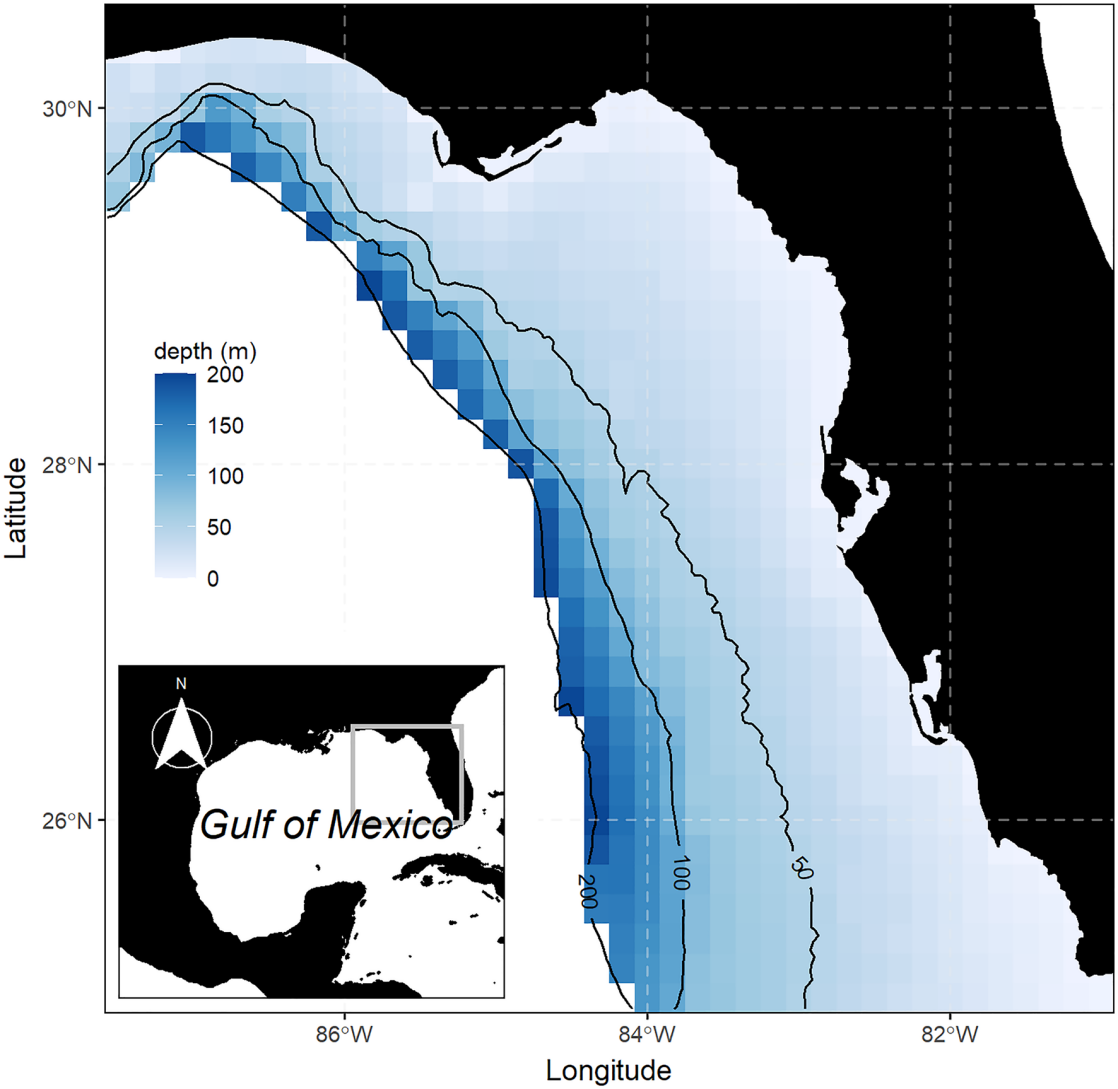
Location of the West Florida Shelf study region. Maps are based on public domain Natural Earth data that were extracted using the ‘*rnaturalearth*’ package^49^ (v. 0.1.0) implemented in R software.

This study was conducted on the WFS that is located in the eastern Gulf of Mexico and extends from the west coast of Florida to about 87.5W longitude. The WFS is a broad shelf and covers approximately 180,000 km^2^, with a depth range between 0 and 200 m^40^ (Fig. 1). In this region, sea surface temperatures vary seasonally and spatially between 32 °C in summer and 15 °C in winter^41^, while salinity fluctuates between 35 and 5 parts per thousand depending on proximity to river outflows. The WFS exhibits high species richness relative to other Gulf of Mexico regions^42,43^, fosters a great diversity of benthic habitat types, including seagrasses and natural reefs^44^, and supports valuable commercial and recreational fisheries^45,46^. However, the WFS region has been impacted by multiple stressors during the last decade: invasive lionfish (*Pterois volitans*), overexploitation, hypoxic events, oils spills and red tides have caused major impacts on species populations, food webs, habitats, and the services they provide^6,47,48^.

To model the WFS ecosystem, we used the EwE software which is recognized as one of the most applied tools to build aquatic and marine ecosystem models^50^. This software suite is composed of three main components: Ecopath, Ecosim, and Ecospace. Ecopath is the static module, which provides a snapshot of the system. It represents the food-web with functional groups that exchange energy through trophic interactions while assuming the mass balance between production and consumption of each functional group. Ecosim is the time dynamic simulation module that allows for predicting ecosystem and species changes over time. It uses differential equations of biomass fluxes and trophic interactions that are modeled based on the foraging arena theory, which assumes that prey move between states of resting or hiding to foraging arenas where they are vulnerable to predation^51^. The Ecosim model usually requires time series of biomass, primary production, and catches or fishing effort in addition to initial parameters inherited from the Ecopath model. Ecosim fitting is achieved by estimating predator–prey vulnerability parameters that define exchange rates of prey biomass from invulnerable states to vulnerable foraging arenas and effectively modulate predator consumption rates and biomass growth as well as mortality of the prey^51^. Finally, Ecospace is the spatial–temporal component of EwE that predicts the spatial biomass dynamics of each functional group over a two-dimensional grid with different spatial properties over time. Ecospace replicates Ecosim dynamics in each grid cell with the addition of equations representing biomass fluxes between neighboring cells. The Ecospace model represents the spatial distribution of fishing mortality using a gravity model in which fishing effort is distributed across cells proportional to the profitability of fishing in those cells, defined as the biomass value of targeted functional groups minus spatial costs that are related to distance to port^38^. The Ecospace habitat capacity model dynamically resolves the suitability of each grid cell for a functional group^52^ based on time-varying environmental conditions. Each grid cell’s habitat capacity is calculated as the product of environmental response functions for multiple environmental factors and/or habitat types. An environmental response function represents the preference of a species to an environmental variable and can take a variety of shapes (e.g. linear, sigmoidal, or dome-shaped). The biomass flux rates between cells are modified depending on the computed habitat capacity in each cell such that movement towards more suitable habitat is favored. The habitat capacity model offers the ability to drive the foraging capacity of functional groups derived from multiple environmental factors. Ecospace incorporates a spatiotemporal framework that allows the inclusion of spatiotemporal data into the ecosystem analysis^53^. The Ecospace model also incorporates an individual-based model (IBM) for a more precise spatial representation of age structure (also known as stanza). The IBM divides each life stage or stanza population into spatial “packets” (or subcohorts) to analyze and predict more accurately the trophic and movement dynamics of multistanza groups^54^. For a detailed description of the EwE software and best practices, see^38,39,55^.

The first version of the WFS EwE model was developed by Okey et al.^56^ primarily to investigate forage fish interactions and was used to evaluate the shading effects by phytoplankton blooms on the marine community. The original WFS model was composed entirely of aggregate species functional groups, and Chagaris et al.^57,58^ later updated the WFS EwE model by adding individual species with age structure in order to integrate with the existing single-species stock assessment and management process. That model version was used to evaluate the ecological and economic tradeoffs of fisheries policy options such as species rebuilding plans, fishing effort reductions, and marine protected areas, as well as impacts of invasive lionfish and changes in primary production^58,59^. In the current study, the WFS model from Chagaris^57^ was further updated by adding new species, splitting functional groups, adding fishing fleets, incorporating more age stanzas for key species, updating time series of biomass and catches used in model calibration, and estimating habitat preference functions from long term fisheries survey datasets. These changes were made based on input from fisheries managers and scientists to better align model outputs with the advice needed for managing fish stocks on the WFS. The current version of the WFS EwE model includes 17 fishing fleets and 83 functional groups (see Supplementary Tables S1, S2 online) and was calibrated to available time series of abundance, catch, fishing mortality, and fishing effort from 1985 to 2020^60,61^.

The WFS Ecospace model encompasses an area ranging from 25 to 30.5 degrees latitude and from − 87.5 to − 81 degrees longitude. It is simulated over a gridded map with a spatial resolution of 10 min (~ 20 km) containing 38 rows × 40 columns from 1985 to 2020 at monthly time steps. To spatially drive the foraging capacity of consumer groups through the habitat capacity model, we considered depth, rugosity, primary production, sea surface temperature (SST), sea bottom temperature (SBT), sea surface salinity (SSS), and red tide for their ability to influence the spatial distribution of marine species. Monthly temperature and salinity maps were extracted from the 3D Gulf of Mexico Hybrid Coordinate Ocean Model (HYCOM)^62^, depth and rugosity maps were obtained from the NOAA National Geophysical Data Center^63^, chlorophyll a concentration data were extracted from the Moderate Resolution Imaging Spectroradiometer (MODIS)^64^, and red tide maps were developed from satellite and in situ HAB sampling (see details below). HYCOM and MODIS datasets were available from 2002 to the present, thus monthly SST, SBT, and SSS raster data from 1985 to 2002 were created by first calculating environmental monthly spatial patterns by averaging available monthly raster data and adjusting that to annual mean trend which were obtained doing linear regression from available data (2002–2020). To hindcast chlorophyll a concentration raster data, we first obtained monthly spatial patterns by averaging available monthly raster data and then applying a deviation from the nutrient concentrations time series^60,65,66^.

Environmental preference functions were obtained by fitting generalized additive models (GAMs) to fisheries-independent data from multiple surveys. Negative binomial and binomial families were investigated using either biomass or presence/absence, but binomial GAMs were selected because they produced more informative environmental response functions that are flexible enough to accommodate trophic processes, as pointed out in previous studies^67,68^. Significant environmental response functions were linked to specific functional groups. For instance, SBT response functions were assigned to demersal and reef-associated functional groups while SST response functions were assigned to pelagic functional groups. Baseline dispersal rates (km·year^−1^) were estimated from movement rates in published tagging studies^69–71^. When dispersal rates were not available, dispersal rates were set based on the general “300-30-3” rule which assumes 300 km·year^−1^ for pelagic, medium, and large reef-associated functional groups, 30 km·year^−1^ for small reef-associated and demersal functional groups and 3 km·year^−1^ for benthic and planktonic functional groups^72^. A two-year spin-up period was applied to all simulations to allow biomass to equilibrate spatially prior to running simulations^73^, while maintaining biomass near the Ecopath starting values. The WFS Ecospace model that served as the starting point for the analysis described here, with initial parameter inputs, is publicly available at the University of Florida’s Institutional Repository^60^ and can be downloaded and run using EwE version 6.6.7 or later.

### WFS Ecospace model stability and calibration procedure

Spatial ecosystem models are rarely validated, calibrated, and assessed in terms of their stability, uncertainty, and ability to reproduce observed patterns, which may hinder their uptake in management^26^. Ecospace models may be unstable with the inclusion of spatiotemporal species interactions and environmental drivers due to misspecification of the vulnerability parameters and environmental response functions. Biomass dynamics in Ecospace models can become unstable because of the spatial mismatch between predator and prey and/or the vulnerability parameters, which may require different values than those estimated in Ecosim. This requires careful inspection of key parameters^52^ and calls for a calibration process to stabilize, validate and fit the Ecospace model.

Ecospace model stability was achieved in a stepwise approach, sequentially adding more complexity while ensuring persistence and a non-exponential increase of FGs over time (see Supplementary Fig. S1) and assuming that species extinctions or exponential increases were not plausible. After achieving a stable fully dynamic model, Ecospace equilibrium biomass maps were validated against the predicted probability of occurrence maps from GAMs for available FGs using a variety of map comparison metrics (see Supplementary Figs. S1, S4). This spatial comparison was intended to highlight FGs that diverged from the occurrence maps and therefore required further inspection.

**Figure 2:**
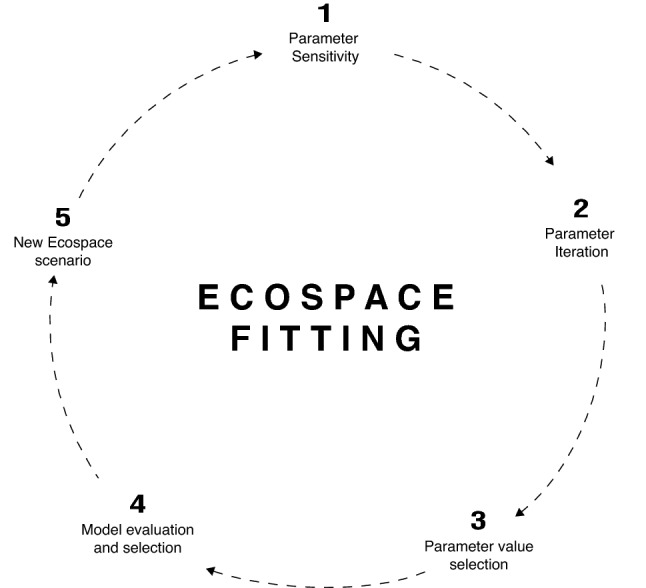
Flowchart of the Ecospace fitting procedure.

Following the stability and validation process described above, a novel fitting routine was developed to identify the most sensitive parameters and their values that reduce bias and improve fits between Ecospace and Ecosim annual biomass, the latter of which were previously fitted to time series data (Fig. 2). We decided to calibrate Ecospace to the Ecosim predicted biomass time series rather than the actual data, which are in a variety of units, to maintain similarity in absolute biomass when moving from Ecosim to Ecospace and because several FGs lacked observed data. We used Ecospace and Ecosim biomass predictions from 1985 to 2001 for comparison since red tides were not included in Ecosim. The calibration process began by changing each vulnerability and dispersal rate parameter, one at a time, while tracking the log-likelihood (LL) and the percent bias (pbias)^74^ between Ecospace and Ecosim. Next, the most sensitive parameters were identified and iterated over 20 different values and those with the lowest total pbias were retained in the model for the next iteration. The iterative process stopped when there was no improvement to the pbias, which occurred after 35 iterations. Each iteration required a total of 2930 Ecospace runs and over 100,000 runs were conducted for the entire procedure. This was achieved using a powerful 48-core workstation computer, running multiple instances of an Ecospace console application in parallel using R (see detailed information about model stability and calibration in Supplementary Information).

### Incorporating red tide effects into the WFS Ecospace model

Red tide events encompass several processes that need to be accounted for when modeling their effects on species and ecosystems including the spatial extent, severity and duration of red tide blooms, their spatial overlap with species of interest, and lethal and sublethal response by species to red tide. The incorporation of red tide effects into Ecospace requires monthly red tide maps for the entire WFS that inform the model about the spatial extent, severity, and duration of such events. Monthly red tide maps were created using satellite imagery to define the possible extent of a harmful algal bloom^75^ and spatial kriging of in situ red tide concentration data from a routine monitoring program^76^. When overlapping with red tides, marine species can react in multiple ways: (i) individuals die, (ii) feeding and growth rate decrease, and (iii) individuals move out of the impacted area. The sublethal responses (feeding, growth, and movement) can be captured using the existing habitat capacity model whereas a new approach for EwE was developed to represent direct mortality from an environmental driver. In both cases we used logistic response functions to model sublethal (feeding and movement) and lethal effects (mortality) as a function of red tide concentrations at each cell and monthly timestep. Multiple red tide response functions were investigated because of the lack of empirical quantitative information on the response of marine organisms to red tides.

#### Red tide mortality response functions

**Figure 3:**
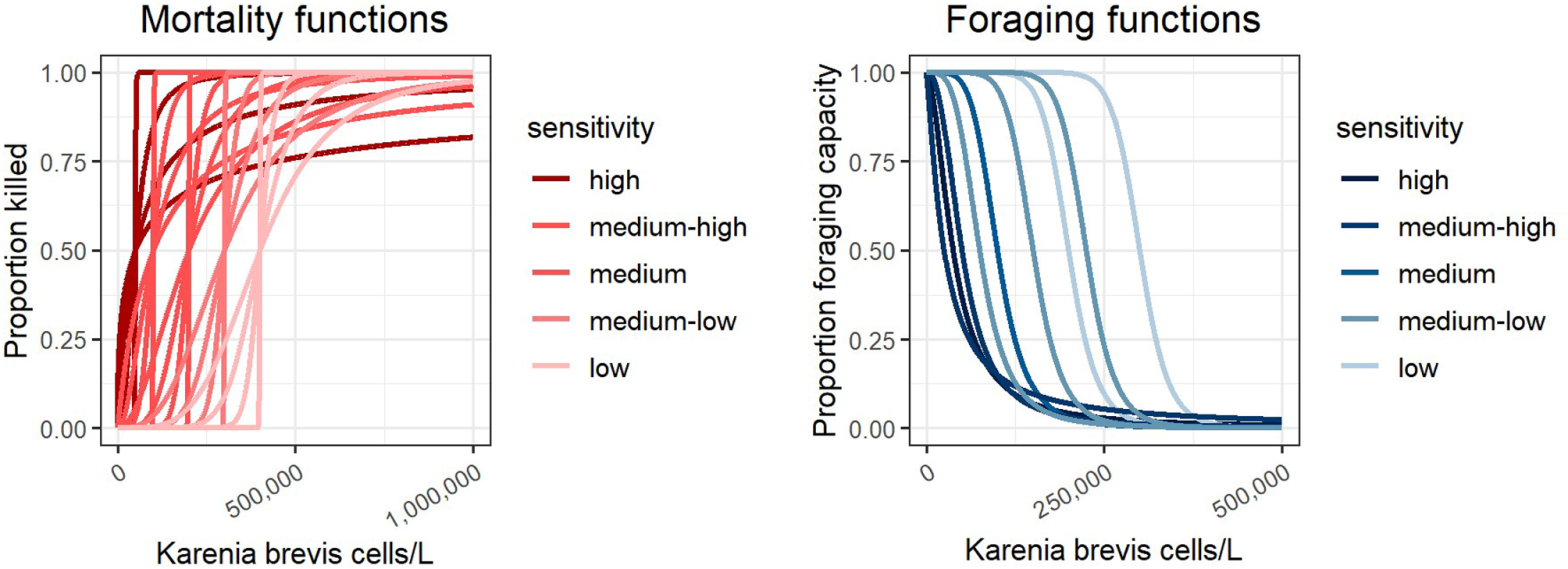
Red tide mortality (lethal red tide effects) and foraging (sublethal red tide effects) response curves used in the WFS Ecospace model, representing multiple sensitivities to red tide. Color lines identify slope steepness and sensitivity. Plots were produced with “*ggplot2*” package^78^ (v. 3.4.0) implemented in R software.

A new mortality response function was created in the EwE software to address mortality due to spatially restricted, episodic perturbations such as red tides. Red tide mortality response functions were applied to the other mortality term (M0) which includes non-predation and non-fishing sources of mortality such as mortality due to disease, old age, or environmental conditions. The red tide mortality response function determined the proportion of biomass killed (*A*) for each functional group (*i*) in each grid cell (*s*) and monthly time step (*t*) as a function of *K. brevis* cell concentration (*x*). The mortality response assumed a logistic curve with a slope (*b*) that was computed from multiple scalar values and multiple inflection points (*c*), representing 20 red tide mortality response functions with multiple sensitivity levels (Fig. 3) and expressed as:

$${A}_{t,s,i}=\frac{1}{1+{(\frac{{x}_{t,s}}{c})}^{-b}}.$$

Inflection points were selected based on the possible negative effects of *K. brevis* concentration on marine fauna and allowed to represent multiple sensitivity levels to red tide events^77^.The proportion killed in each cell and timestep was converted to an annual instantaneous mortality rate, $\widehat{A}$, and scaled to the Ecopath other mortality rate, *M0*_*base*_ (also an instantaneous rate), to return the other mortality multiplier term, *M0*_*mult*_, for each functional group, grid cell, and monthly time step:

$${\widehat{A}}_{t,s,i}=-\mathrm{ln}(1-{A}_{t,s,i})\cdot 12,$$

$${M0mult}_{t,s,i}=\frac{{\widehat{A}}_{t,s,i}}{{M0base}_{i}}.$$

The biomass loss due to red tides for each species, grid cell, and timestep was calculated as the product of the proportion killed, ${A}_{t,s,i}$, and the biomass in that cell ${B}_{t,s,i}$. An annual index of red tide mortality was computed for each functional group, *i*, by summing the biomass loss from red tides over all months and cells within a year and dividing by the average annual biomass across the entire model domain.

#### Red tide sublethal response functions

The Ecospace habitat capacity model was used to model sublethal responses on specific functional groups in two ways. First, functional responses to algal concentrations reduce a group’s habitat capacity (i.e. the foraging arena size) in affected grid cells, which thereby reduces consumption and biomass growth in areas impacted by red tides. The reduced foraging capacity in an affected cell will increase the movement rate out of that cell. This allows mobile species to avoid red tide blooms and may mitigate direct mortality losses if cells with suitable habitats are nearby. The red tide foraging response was defined using a logistic curve that decreased with red tide cell concentrations. The foraging responses had lower inflection points than the mortality responses since sublethal effects and avoidance responses are likely to be experienced at lower *K. brevis* concentrations, prior to mortality^79^. Three foraging response curves were generated for each mortality inflection point *c*, at multiple sensitivity levels (25, 50, and 75%) of the mortality inflection point. The slope for each foraging response curve was scaled relative to the inflection point (using a multiplier of -5·10^–5^) such that the shape of the curve did not change, while its position on the x-axis varied. This resulted in 15 foraging response functions (a high, medium, and low foraging response function for each of the 5 mortality sensitivity levels), that represent multiple sensitivity levels (Fig. 3).

### Red tide scenarios

A total of 160 Ecospace scenarios representing different sensitivities and combinations of response functions were run (see Supplementary Table S3 online). The first 20 only included the mortality response functions (five sensitivities with four slopes each), while the next 60 included three foraging responses for each of the 20 mortality responses representing the different sublethal sensitivities, relative to each mortality curve. This allowed us to investigate the effects of direct lethal and sublethal red tide effects. To tease apart specific red tide effects from indirect effects arising through trophic interactions, the 80 configurations were run once with red tide response functions applied to all consumer groups and then again with response functions applied to one species of management interest, gag grouper (see Supplementary Table S3 online).

To run such scenarios, we used the Ecospace Console Application which is a command-line version of the Ecopath with Ecosim software that runs a non-user interface version of the Ecospace model that can be parameterized and executed via common code-based analytical software such as R or Python. Bypassing the user interface allowed us to standardize and automate the process and run the simulations in parallel on a large workstation computer (48 cores/96 threads). This approach increased efficiency and eliminated the potential for errors related with manually adjusting response function parameters.

### Output metrics for red tide effects

For each red tide scenario, annual time series and monthly geospatial data were extracted from 1985 to 2020. This included for each functional group, maps of biomass (t·km^−2^) and biomass loss due to red tides (t·km^−2^). Some geospatial outputs were annually averaged over all grid cells to obtain a time series value for the entire spatial domain. Red tide effects were assessed on the ecosystem as well as a single species, gag grouper. We selected gag grouper as a case study because of its importance to commercial and recreational fisheries and its last stock assessment included red tide effects^24^.

#### Ecosystem impacts

Ecosystem impacts were evaluated by using the 80 scenarios that applied red tide effects to all consumer groups (see Supplementary Table S3 online). Four ecological indicators (biomass and catch-based) were investigated to describe the ecosystem effects of red tides. Biomass-based indicators may be useful to describe red tide effects across different components of the ecosystem^28^ whereas catch-based indicators reflect the impacts on fisheries^80,81^. We included three biomass-based indicators: total biomass of species (B_T_) (t·km^−2^), biomass of harvested (commercial and recreational) species (B_har_) (t·km^−2^), and biomass loss due to red tide (B_loss_) (t·km^−2^) and one fishery indicator as the total catch (C_T_) (t·km^−2^·year^−1^) summed over all species and fleets. To summarize the impacts of red tide on the marine community, functional groups were further aggregated according to ecological traits which were based on habitat and taxonomy. Ecological traits groups were upper trophic level (TL) pelagic, lower TL pelagic, demersal, reef-associated, and benthic invertebrates while taxonomic groups included marine mammals, elasmobranchs, fishes, and cephalopods (see Supplementary Table S1 online). Juvenile FGs were not included in the marine community analysis due to confounding effects associated with contrasting habitat preferences with adult individuals. Predicted ecosystem and community indicators were represented distinctly for scenarios including only lethal red tide effects and scenarios including lethal and sublethal red tide effects to tease apart the effects of each response type.

#### Population impacts

We evaluated the impacts of red tide on a single population, gag grouper. First, we screened the 160 scenarios comparing the predicted total red tide loss rate (combined over all ages) to the 2005 red tide loss rate of 0.77 estimated by the most recent stock assessment^24^. We also calculated root mean-square error (RMSE) between the Ecospace predicted biomass, adjusted for selectivity, to observed fisheries dependent and independent indices used in the stock assessment. We defined acceptable scenarios as those which predicted a 2005 red tide loss rate value within 2 standard deviations of the estimate from the stock assessment (0.77 ± 0.12). Acceptable scenarios were also considered only if RMSE between predicted and observed time series fell within 10% of the lowest RMSE. We differentiate between runs that applied red tide effects to all consumer groups or only gag, as indirect and direct red tide effects, respectively (see Supplementary Table S3 online). This approach allowed us to disentangle food-web red tide effects (red tide effects applied to all consumer groups) from specific red tide effects (red tide effects applied only to gag grouper ).

### Validating predicted effects of red tide

We attempted to validate predicted red tide effects by comparing the percent of change in terms of each marine organism’s abundance and biomass inside versus outside and before and after a past red tide bloom. This analysis required matching sampling locations before, after, and during several red tide events. However, the validation datasets (or empirical data) are often limited because of the spatial and temporal coverage of fisheries surveys, such as a random sampling design in which sampling stations are sparsely dispersed or only occur seasonally. Therefore, we validated the Ecospace model predictions by comparing mean densities in/out and before/after three red tide events where fish abundance data were available to do so. First, red tide events were spatiotemporally matched with empirical observations from multiple fisheries independent surveys and three red tide events and surveys were matched for this comparison. This included the Florida Fish and Wildlife Conservation Commission’s baitfish cruise trawl survey for April 2005 (before the red tide event) and November 2005 (after the red tide event), the SEAMAP^82^ bottom trawl for June 2015 (before the red tide event), and October 2015 (after red tide event) and the NOAA National Marine Fisheries Service (NMFS) bottom longline survey for August 2014 (inside/outside red tide event). For the trawl surveys, the observed red tide effect was calculated by comparing the average abundance of marine organisms between both months in the region where the red tide event took place. For the bottom longline, the observed red tide effect was calculated by comparing the average abundance of marine organisms inside and outside the red tide affected area. To compare calculated and observed values, we first selected Ecospace output raster data of the most abundant functional groups caught in each survey. Monthly raster data of selected functional groups were summed up and clipped using a polygon created from sampling stations from each survey. Mean and standard error values were calculated for each computed raster.

## Results

### Ecosystem impacts

**Figure 4:**
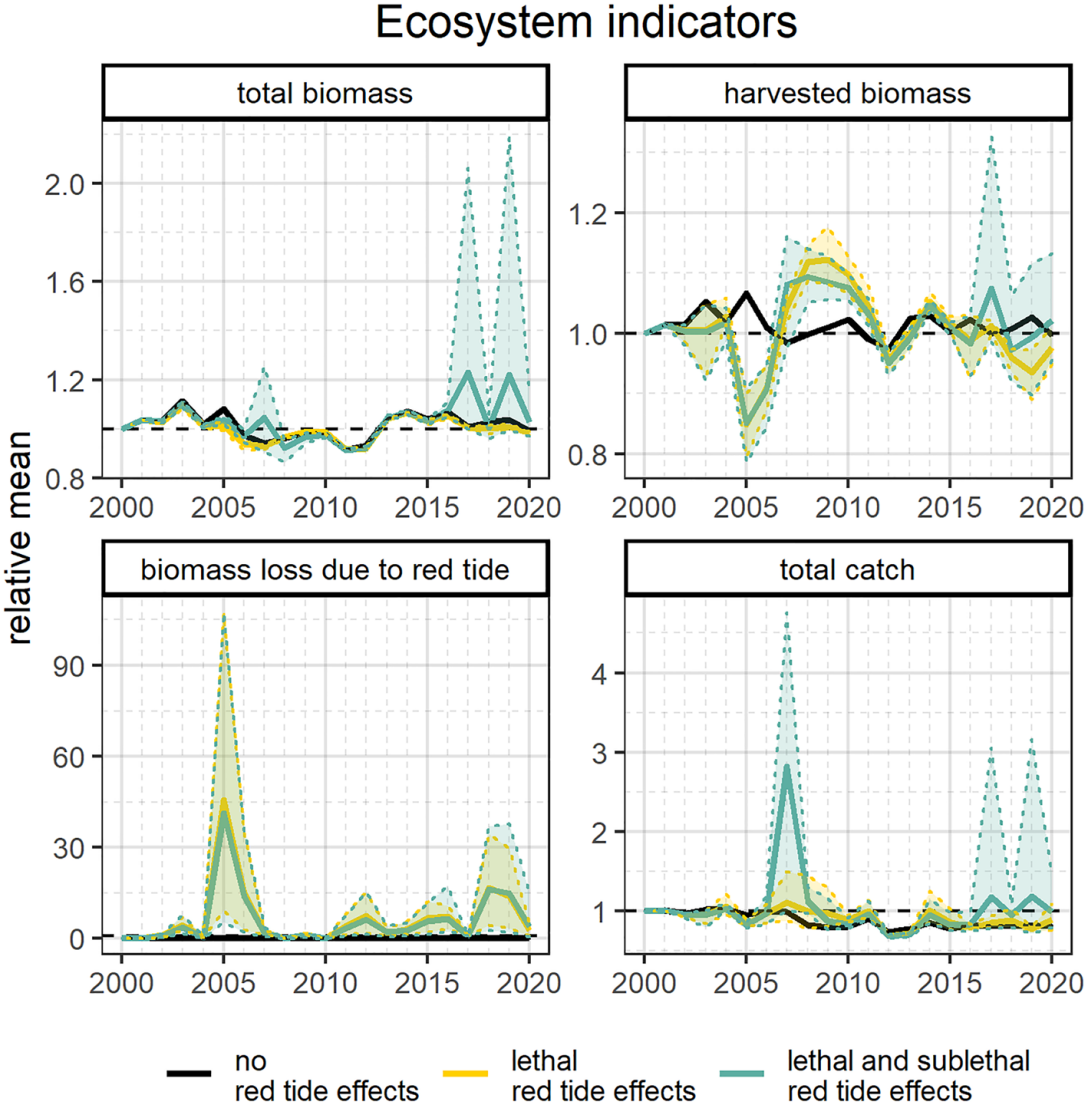
Predicted time series of relative ecosystem indicators: total biomass (B_T_), harvested biomass (B_har_), biomass loss due to red tide (B_loss_), and total catch (C_T_). Shadows represent 95% confidence intervals based on the multiple simulated scenarios. Black solid lines represent the scenario without red tide effects. Yellow solid lines and shadows represent scenarios (20 scenarios) including lethal red tide effects. Blue solid lines and shadows represent scenarios (60 scenarios) including lethal and sublethal red tide effects (detailed scenario information is included in Table S3 online). Plots were produced with “*ggplot2*” package^78^ (v. 3.4.0) implemented in R software.

Ecosystem indicators showed biomass and catch fluctuations over time and differences when considering red tide mortality response and/or sublethal foraging responses (Fig. 4). B_T_ showed similar trends among all three red tide effects configurations and increased from 2000 to 2005, decreased from 2005 to 2012, and recovered in 2013. When red tide effects were not considered, B_T_ was higher in 2005. When applying sublethal red tide effects, B_T_ increased in 2007, 2017, and 2019 that was driven by the jellyfish group. Similar to B_T_, when no red tide effects were included, B_har_ increased in 2005. If red tide effects were applied, B_har_ dropped considerably in 2005 but recovered by 2008. Such biomass changes were not captured when red tide effects were not included. The inclusion of sublethal effects showed higher B_har_ in 2017 due to the greater increase in large crab biomass. B_loss_ showed only positive values when including red tide effects and the highest levels of biomass loss due to red tide were found in 2005 followed by 2018, 2019, 2006, and 2012. C_T_ trends were similar among red tide effects configurations except for 2007, 2017, and 2019 when the inclusion of sublethal red tide effects led to higher C_T_ estimates that were driven by the biomass increase in the large crab group.

**Figure 5:**
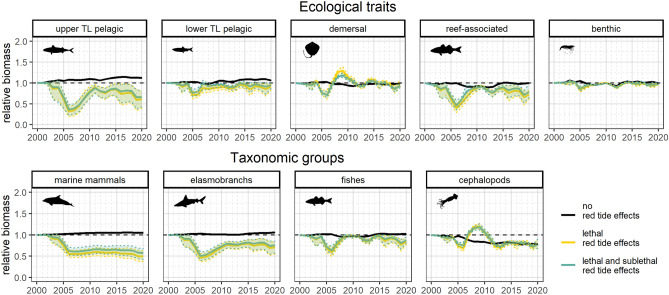
Predicted time series of community indicators: relative biomass for ecological traits and taxonomic groups. Shadows represent the 95% confidence intervals based on the multiple simulated scenarios. Black solid lines represent the scenario without red tide effects. Yellow solid lines and shadows represent scenarios (20 scenarios) including lethal red tide effects. Blue solid lines and shadows represent scenarios (60 scenarios) including lethal and sublethal red tide effects (detailed scenario information is included in Table S3 online). Plots were produced with “*ggplot2*” package^78^ (v. 3.4.0) implemented in R software. Silhouettes were downloaded from phylopic database (http://phylopic.org/).

Community results showed predicted biomass fluctuations over time for all groups when red tide effects were included and the exclusion of red tide effects showed more constant biomass trends (Fig. 5). When including red tide effects, ecological traits and taxonomic groups showed biomass decreases in 2005 and 2006 followed by recovery in the following years. Upper TL pelagic, reef-associated, marine mammals, elasmobranchs, and fishes showed the greatest biomass reductions. Demersal and cephalopods groups displayed the highest recovery after biomass depletion of predators, while marine mammals and elasmobranchs did not show recovery after 2005. When including sublethal red tide effects, most groups showed higher biomass levels.

### Population impacts

**Figure 6:**
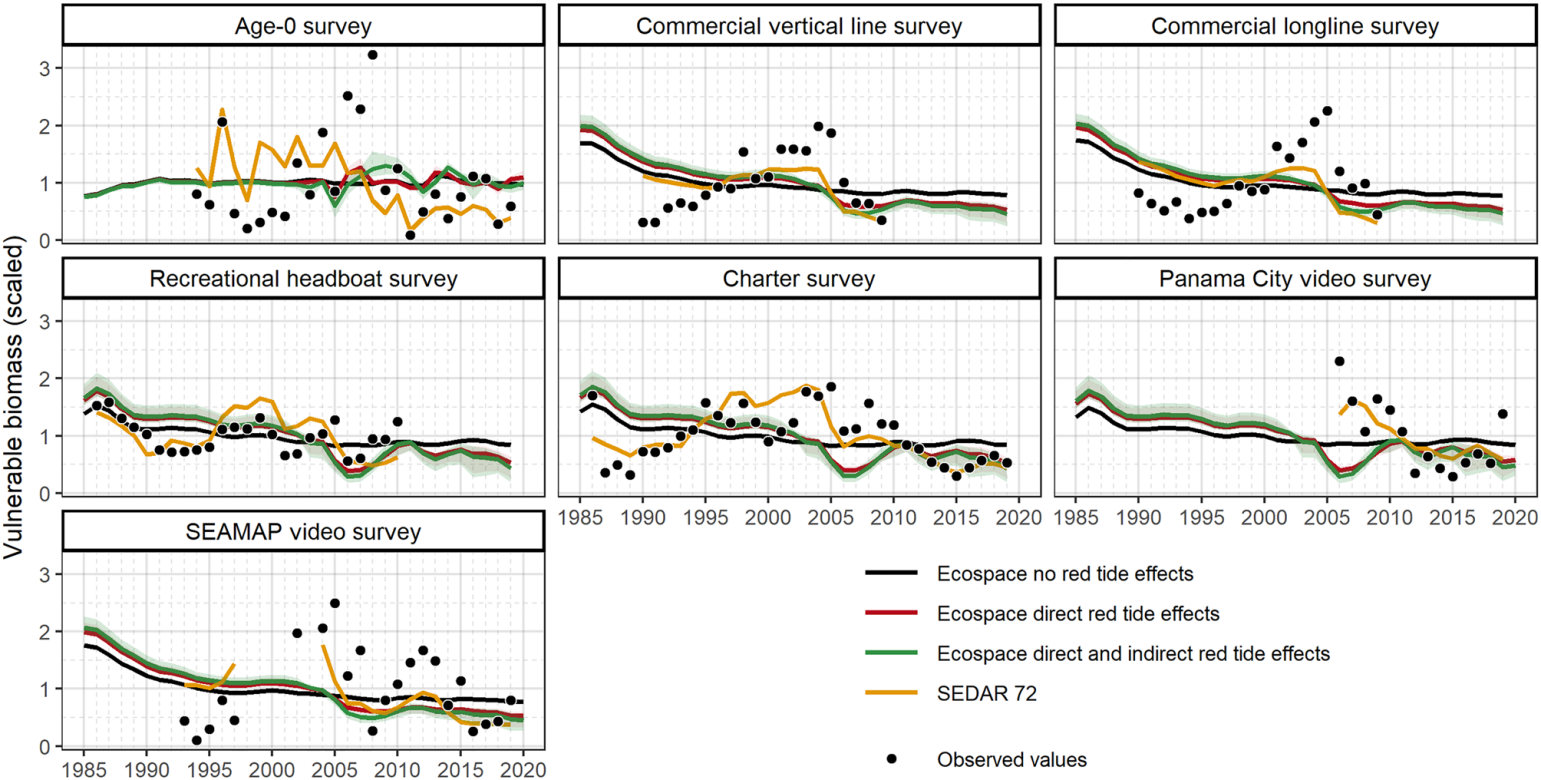
Mean gag vulnerable biomass predictions from validated Ecospace direct effects and direct and indirect (food-web) red tide effects scenarios (red and green line, respectively), Ecospace without applying red tide effects (black line), and SEDAR 72 (yellow line) overlaid on the observed index of abundance values (points) taken from the SEDAR 72 assessment. Shadows represent the 95% and 5% confidence intervals. Plots were produced with “*ggplot2*” package^78^ (v. 3.4.0) implemented in R software.

**Figure 7:**
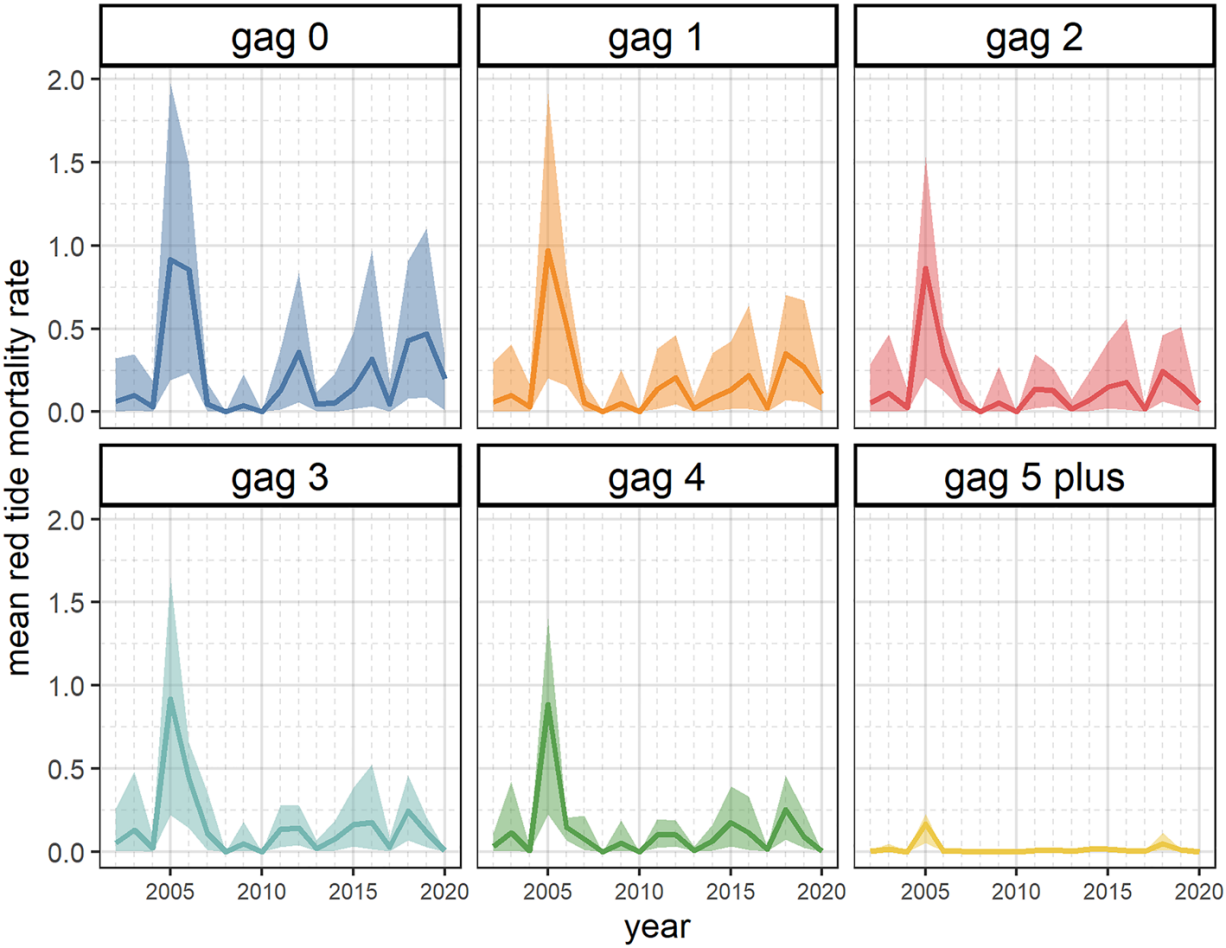
Time series of estimated mean and 95% confidence interval red tide mortality (year^–1^) for gag from 2002–2020 generated by the WFS Ecospace model. Colored lines and shades represent estimates for 71 selected scenarios (for detailed information on red tide scenarios see Table S3 online). Plots were produced with “*ggplot2*” package^78^ (v. 3.4.0) implemented in R software.

To summarize the population effects on a single stock, gag grouper, we retained 71 red tide scenarios (48 applying direct red tide effects only and 23 applying direct and indirect food-web effects) out of 160 based on RMSE and 2005 mortality criteria (see Supplementary Figures S5, S5 online). From these scenarios, gag grouper biomass indices results showed differences among Ecospace model configurations (Fig. 6). Predictions from the Ecospace models including red tide effects showed a reasonably good fit with the observed values and produced similar biomass trends to the SEDAR 72 stock assessment model. In contrast, the Ecospace model predictions without red tide effects did not capture the variability in gag grouper biomass. The predicted differences between the Ecospace model with and without red tide effects were more noticeable during the period that red tide data were available, from 2002 to 2020. All vulnerable biomass indices showed a decrease in 2005 due to high red tide mortality rates, especially in juvenile age stanzas (Fig. 7). The biomass decline in 2005 was only greater when indirect food-web effects of red tide were included. After the 2005 decline, the Age-0 survey showed a recovery that was more prolonged when applying food-web effects. The inclusion of food-web red tide effects into the Ecospace model resulted in higher biomass for age-0 gag in the years following the 2005 red tide. The estimated red tide mortality for gag grouper showed fluctuations over time with the highest values in 2005 followed by 2012, 2016, 2018 and 2019 (Fig. 7). Red tide mortality rates were higher for younger gag grouper stanzas, except for 2005 when red tides caused a similar mortality rate among all age stanzas.

### Model validation

**Figure 8:**
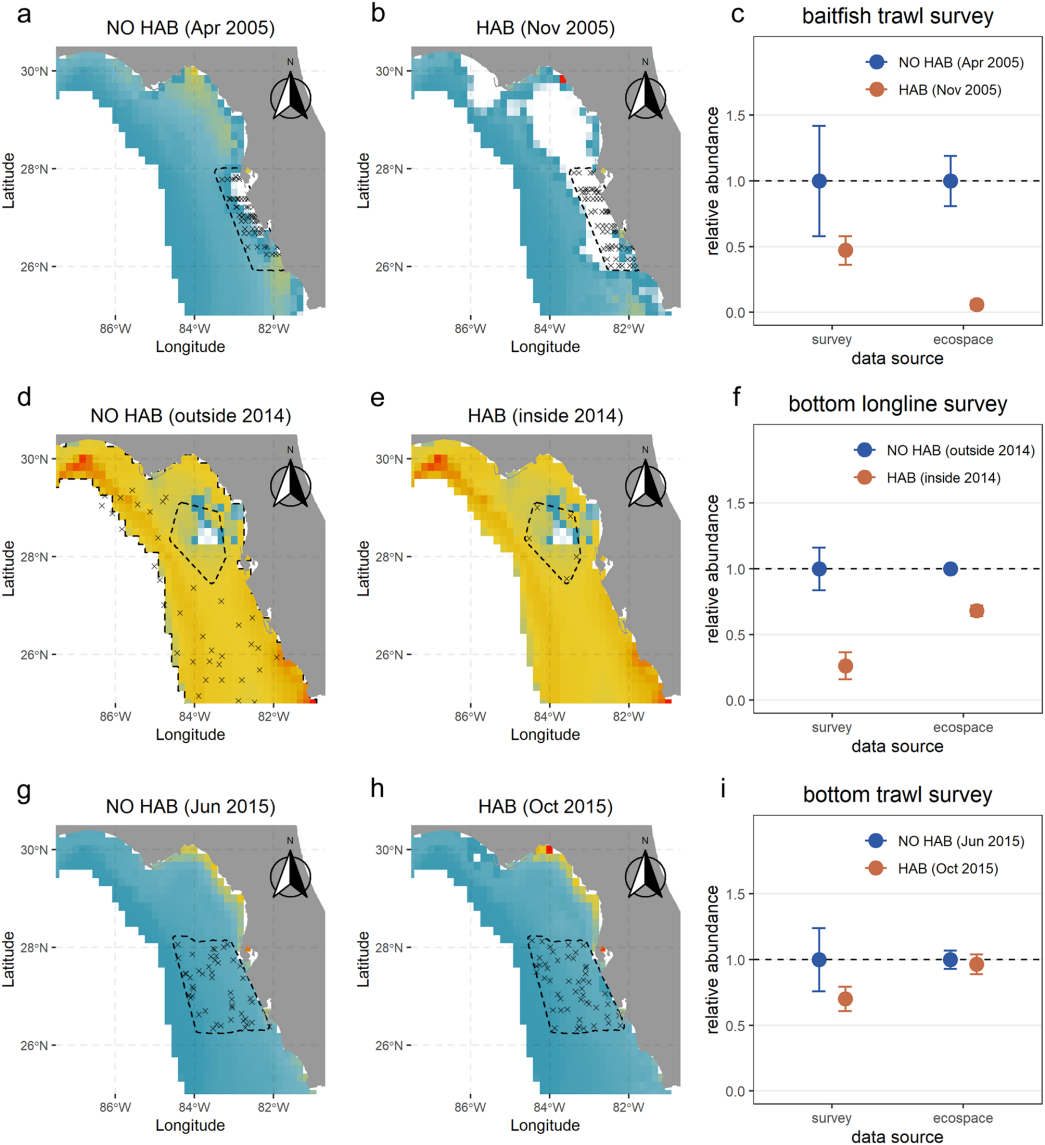
Ecospace (predicted) data before and after Harmful Algal Blooms (HABs) and outside and inside impacted areas for multiple surveys data: (**a–c**) baitfish trawl, (**d–f**) bottom longline survey and (**g–h**) bottom trawl and mean relative abundance of marine organisms and 95% confidence interval of Ecospace and each survey (observations) at represented time steps. Red points represent abundance values prior to or outside HABs events and blue points represent abundance values during or inside HABs events. Maps represent the Ecospace predicted abundance of FGs for each survey and each selected time step. Map color intensities represent abundance value (white: zero; blue: low; red: high). Plots were produced with “*ggplot2*” package^78^ (v. 3.4.0) implemented in R software. Maps are based on public domain Natural Earth data that were extracted using the ‘*rnaturalearth*’ package^49^ (v. 0.1.0) implemented in R software.

The red tide impacts were obvious at multiple ecological levels in terms of magnitude and spatiotemporal scale, especially in specific months and years. The differences in biomass before/after and outside/inside predicted by Ecospace were greater or less than those observed in the survey data (Fig. 8). All three study cases (2005, 2014, and 2015) displayed abundance decreasing after red tide events and inside impacted areas and the effect on aggregate fish abundance were more evident in the years 2005 and 2014 than in 2015. Spatiotemporally, abundance loss was spread over a large area in 2005 while the HABs were more localized in the central region of the study area. The Ecospace model overestimated biomass loss from red tide in 2005, while it underestimated biomass loss due to red tide in 2014 and 2015.

## Discussion

This study represents the first attempt to incorporate spatially restricted episodic natural mortality events into an ecosystem model through response functions affecting mortality, foraging, growth, and movement of marine organisms. Despite the spatiotemporal complexity of such events, the WFS Ecospace model was able to assess red tide effects at multiple ecological scales and produce a time series of red tide mortality for an important managed species, gag grouper. Sublethal and indirect food-web red tide effects triggered compensatory responses such as avoidance behavior, predation and/or competition release. The inclusion of a comprehensive validation, uncertainty assessment, and calibration in the WFS Ecospace model increased its robustness and management utility.

According to our analysis, red tides have had a major impact in the WFS region at the ecosystem, community, and population levels, and the year 2005 was highlighted as the most severe red tide event over the period considered. Ecosystem and community indicator results showed a reduction in relative total and harvested groups biomass and relative total catch in years with major red tide events, especially in 2005, which may suggest ecosystem degradation and fishery impacts during severe events. The 2005 red tide is still recalled as the most extreme red tide of recent years, in terms of persistence, spatial extent, and severity that affected socioeconomic^83^ and ecological^84^ aspects of the system, although perceptions may be biased^85^. Red tide effects are very dependent on the spatiotemporal distribution of both red tide events and species. In 2005, red tide extended offshore and both juvenile and adult gag grouper suffered high and similar red tide mortality rates. Currents and favorable environmental conditions may induce the propagation of red tides to offshore adjacent regions similar to previous red tide events in the WFS^15^. The 2005 red tide was also highlighted as having a high spatial overlap with juvenile and adult red grouper^86^.

In most years, red tides remained close to shore and negative effects were more important for species inhabiting this coastal area of the WFS region. In 2006, 2012, 2018 and 2019, younger gag grouper stanzas were impacted more than adult stanzas because of the spatial overlap with red tide events that occurred nearshore. Therefore, red tide is a potential driver of young-of-year survival and year class strength of gag grouper, and recruitment may be lower when considering red tide information into stock assessment models^87,88^. The Ecospace model used fishing, food-web, and red tide effects to explain population trends, while single-species stock assessment models depend on fishing mortality and recruitment deviations to capture changes over time. Ecospace predictions of gag biomass were similar to those predicted by the stock assessment model, which may point to stock assessment model misspecification where recruitment deviations are used to account for changes that are actually caused by time-varying natural mortality that is unaccounted for in the stock assessment model.

Predicted red tide effects differed across species and functional groups due to production and total mortality rates, dispersal rates, the spatial overlap between red tides and preferred habitat, and trophic interactions. For example, the higher production to biomass ratio of invertebrates’ groups allowed them to recover faster in the model, and community metrics indicated different recovery rates between lower trophic levels (e.g. lower TL pelagic and benthic) and higher trophic level groups (e.g. upper TL pelagic, marine mammals, and elasmobranchs). In line with these results, an ecosystem model study^89^ investigated the effect of perturbations in the food-web and demonstrated faster recovery of benthic invertebrates than fish groups due life history traits and the number of trophic links.

Previous attempts to assess red tide impacts on the WFS ecosystem used non-spatial EwE models of the system that required the input of red tide removals via a pseudo-fishing fleet and a red tide severity index trend as an effort forcing time series^27,28,90^. By specifying the baseline red tide removals and a severity index trend in EwE, the mortality rates are thereby forced by the user and not an emergent property of the model. In this study, red tide effects and biomass loss emerged from the overlay of red tide blooms with species distributions and the application of the various logistic response functions. The level of spatiotemporal overlap between stressors such as red tides and marine species has a critical influence on their impacts^91^. Our approach provides a notable improvement in the representation of red tides and consequently a more accurate evaluation of red tide effects.

The inclusion of red tide lethal effects was essential to comprehensively assess red tide impacts. The novel mortality response function applied to the *M0* term allowed the representation of perturbations such as red tides. This new feature can be applied to other episodic natural mortality events that require a spatiotemporal ecosystem modeling framework. For instance, oils spills, cold kills, and ocean heat waves can cause high mortality rates on marine fauna that may be better evaluated by using a spatiotemporal modeling approach that applies mortality response functions. The incorporation of species-specific mortality response functions may also help to explore species vulnerability to episodic perturbations.

Sublethal red tide effects were incorporated as foraging response functions into the habitat capacity model inside Ecospace^52^ that represents a decrease in habitat quality as a function of red tide concentrations. When sublethal red tide effects were included, the WFS ecosystem model predicted greater and faster recovery of total biomass and catch after a severe red tide year, as species were allowed to move away from impacted areas. Active spatiotemporal avoidance of organisms to stressors has been widely documented in the marine realm^92–94^. In addition, red tides can decrease habitat quality and cause habitat fragmentation leading to emigration of marine populations and local depletion^95^. In WFS Ecospace, avoidance was captured through the habitat-capacity model, rather than by modifying dispersal rates directly, and the sublethal effects were assumed to be experienced at lower concentrations than those that would result in mortality. This allowed groups to potentially move before they are killed, resulting in a net reduction in biomass loss from red tide. However, this compensatory behavior can be hindered under repeated or prolonged exposure to red tides^96^ or if a red tide is so expansive that organisms cannot find unaffected habitats nearby. The ability of groups to actively avoid disturbed regions is influenced by their baseline dispersal rates, whereas groups with high dispersal rates are expected to experience fewer effects of red tide. However, our results suggested that dispersal rates were not that important because highly mobile and upper TL pelagic group groups (e.g. marine mammals) did not show differences when including sublethal effects. This may suggest that sublethal effects were not strong enough to disperse organisms out of impacted areas or movement could not compensate for low production rates of some FGs or misspecification of dispersal rates, vulnerability parameters, and/or response functions by the fitting procedure.

Compensatory responses also emerged from the model as a result of food web related processes. The rapid recovery of demersal and cephalopod groups may suggest predation and/or competition release that allow faster biomass growth for certain species following red tide impacts. In addition, the increase in biomass of age-0 gag following the 2005 red tide only occurred in model runs where all consumers were impacted by red tide, suggesting a trophic-driven compensatory response likely due to competition release. Several studies reported trophic cascades after the impact of stressors^59,97–99^. It is recognized that red tides impact the whole marine community^100^ and therefore alter natural mortality and trophic interactions. Other WFS ecosystem models demonstrated a trophic cascade as red tides caused biomass increase for piscivores, planktivorous, and detritivores groups but biomass reductions for invertebrate eaters and omnivores groups due to changes in predation mortality^27^. This study also indicated that mortality due to red tide may result in reduced competition because of top-down effects. By evaluating red tides within a trophic dynamic model, food-web effects such as reduced competition and predation may allow for compensation of certain forage groups or juveniles for a brief period of time following red tide events, which could lead to a more resilient system.

One of the main sources of uncertainty in estimating red tide effects was the application of the same red tide response functions across all consumer functional groups in each model run. Responses to stressors most likely vary among taxa because of genetic, physiological, and behavioral traits^96^. However, we did not investigate different combinations of red tide response functions to different species or functional groups because studies of species-specific responses to red tide concentration are lacking and the computational requirements to do so would be prohibitive. Performing laboratory experiments and monitoring the marine community behavior before, during, and after red tide events would help to specify appropriate, taxa specific, red tide response functions and improve model predictions.

Another source of uncertainty specific to modeling red tide impacts is related to our limited ability to map red tide severity and subsequent effects on water quality over the entire WFS. Satellite estimates may be biased because of meteorological events (water turbidity and clouds), the fine-scale patchiness of red tides, and the inability to detect subsurface blooms^101^. The in situ* K. brevis* concentration data can also be biased due to opportunistic sampling and limited spatiotemporal coverage of the . Hypoxic zones tend to form in association with red tides^102^, however this effect was not included in the model due to the lack of dissolved oxygen data needed to generate the monthly driver maps for Ecospace. Ecospace predicts biomass dynamics over a two-dimensional grid which limits the ability to capture the vertical dynamics of red tides. Other spatiotemporal ecosystem model tools such as Atlantis may help to fully represent red tide dynamics in a three-dimensional space^103^. In addition, the red tide severity maps could be improved upon using other methods such as ocean circulation models, spatial delta-generalized linear mixed models, co-kriging and physical-biogeochemical models that represent harmful algal blooms dynamics across depth^13,104–106^. Enhanced monitoring of red tides and dissolved oxygen through remote sensing, citizen science, and a systematic and fine-scale sampling design could greatly improve our ability to capture red tide effects in the WFS Ecospace model. Despite the uncertainties associated with surface water properties derived from satellites and the in situ data, particularly in coastal waters, these data represent the best scientific information available and they are regularly used to monitor and track red tide events. In addition, environmental factors such as temperature nutrients, and CO_2_ may influence bloom toxicity^107–109^, and additional studies are needed to understand these relationships.

We were limited in our ability to validate impacts of past red tides on fish populations due to the lack of data specific for these events. Fishery independent monitoring programs on the WFS were primarily designed to track overall abundance trends for single species and were not intended to measure localized mortality events, such as red tides, that are restricted in both space and time. In our study, only three red tides (2005, 2014 and 2015) were able to be matched with fish monitoring data collected either before/after or inside/outside a red tide event. The validation process highlighted that the WFS Ecospace model overestimated red tide effects in 2005 but underestimated effects in 2014. This calls for targeted fisheries independent and comprehensive ecosystem monitoring in association with red tide sampling in routinely impacted areas such as southwest Florida, and in occurrence with red tide blooms. Considering that HABs are expected to increase in frequency and severity in the future^110^, a comprehensive understanding of red tide effects on the ecosystem, community, and population levels is key to achieving management objectives under an ecosystem-based fisheries management (EBFM) framework. The model was also limited in its ability to represent the finer scale temporal and spatial dynamics of red tide events. Red tides are highly dynamic, and blooms may form and dissipate within a few weeks, whereas Ecospace estimated impacts at a monthly timestep, over a 10-min spatial grid. Moving to a higher spatial resolution (down to ~ 4 km) is achievable given available spatial–temporal driver data but would drastically increase model run time. Representing faster time dynamics of red tide is limited by our ability to develop synoptic daily or weekly red tide maps and would require additional software development to run the ecosystem model at a finer timestep.

The episodic increase in mortality from red tides can impact stock status determination and management advice for exploited fish stocks, Our results quantified the mortality caused by red tides on the gag grouper population, which was declared to be overfished in 2009 and again in 2021. Incorporating red tide effects is a priority for Gulf of Mexico gag grouper stock assessments, as dictacted by Terms of Reference that outline requirements of the assessment process. The estimated age-specific red tide mortality rates for gag grouper generated by the WFS Ecospace model were included in a sensitivity run of the 2021 gag grouper stock assessment^24^. In 2021, the Gulf of Mexico Fisheries Management Council convened its Scientific and Statistical Committee (SSC) to provide catch advice for gag grouper based on projections from the stock assessment. At that time, a severe red tide was ongoing and the WFS and the catch projections were sensitive to the assumed red tide mortality rate. The WFS Ecospace model was quickly updated and rerun, and red tide mortality was estimated through October 2021 at about a 2-week lag. The contemporary estimates of red tide mortality were deemed best scientific information available by the SSC and used to configure the final catch projections recommended to the Council. Hence, the WFS Ecospace model can track red tide impacts at a near real-time resolution by coupling in situ HAB samples with remote sensing platforms^111^. This demonstrates how a red tide mortality rate index with associated uncertainty generated by a spatiotemporal ecosystem model can inform and enhance stock assessments, provide advice for management, and move towards achieving EBFM^87,112,113^. Operationalizing red tide predictions may increase the ability of fisheries managers to respond more effectively and be more proactive to these events. In addition, a user-friendly and open-access shiny application^114^ named “*redtideVIS*” (https://redtidevis.shinyapps.io/redtideapp/) was developed to easily visualize biomass and red tide mortality rate predictions and associated uncertainty in the WFS ecosystem to facilitate their use in management.

To our knowledge, this study represents the first attempt to incorporate periodic environmental perturbations into a spatiotemporal ecosystem modeling framework. Results suggest severe red tide impacts have occurred on the WFS at the ecosystem, community, and population levels in terms of biomass and catch. Importantly, this work presents a novel mortality response function that represents the lethal effects of perturbations, proposes a novel Ecospace model calibration process, and presents a concrete example of how ecosystem information can be quantified for use in single-stock assessment models^113^. This study also represents a step forward to operationalize spatiotemporal ecosystem models for management purposes, evaluate the cumulative effects of fishing and red tide mortality on fish populations, and forecast the effects of unprecedented severity and coverage of future red tide events^8^. Furthermore, the approach could be adapted to other spatiotemporal stressors like oil spills, cold kills, and ocean heat waves.

## Supplementary Information

Supplementary Tables.

Supplementary Information.

## Data Availability

The EwE model is publicly available at the Ecobase repository (http://ecobase.ecopath.org/). The entire model database that served as the starting point for the analysis described here has been archived on the University of Florida’s Institutional Repository (http://ufdc.ufl.edu/IR00011604/00001). The WFS data package is accessible from the National Centers for Environmental Information (https://doi.org/10.25921/t26e-wj91).
