# Supplementary material for: Evaluating red tide effects on the West Florida Shelf using a spatiotemporal ecosystem modeling framework
Source: Sci Rep. 2023 Feb 13;13:2541. doi: 10.1038/s41598-023-29327-z (PMC9925760; doi:10.1038/s41598-023-29327-z)
Supplement: Supplementary file 2 — Supplementary Information. [file 41598_2023_29327_MOESM2_ESM.docx]

**Supplementary Information**

EVALUATING RED TIDE EFFECTS USING A SPATIOTEMPORAL ECOSYSTEM MODELING FRAMEWORK

Daniel Vilas^1,2,*^, Joe Buszowski^3^, Skyler Sagarese^4^, Jeroen Steenbeek^3^, Zach Syders^1^, David Chagaris^1,2, *^

^1^Fisheries and Aquatic Sciences Program, School of Forest Resources and Conservation, University of Florida, Gainesville, FL 32611, United States;

^2^Nature Coast Biological Station, Institute of Food and Agricultural Sciences, University of Florida, Cedar Key, FL 32625, United States;

^3^Ecopath International Initiative, Barcelona, Spain

^4^NOAA Fisheries Service – Southeast Fisheries Science Center, Miami, FL, 33149, United States

*** Corresponding author email:** David Chagaris [dchagaris@ufl.edu](mailto:dchagaris@ufl.edu); Daniel Vilas [danielvilasgonzalez@gmail.com](mailto:danielvilasgonzalez@gmail.com)

**Keywords:** red tides, harmful algal blooms, ecosystem, Ecospace, West Florida Shelf

## Supplementary WFS Ecospace Model Stability and Calibration Procedure

Model stability was checked at each step by sequentially adding model complexity in terms of the incorporation of environmental variables that drives the foraging capacity of FGs, going from a static to a fully spatially dynamic Ecospace model. The first step included historical fishing effort time series, static depth, and static rugosity as environmental variables (*static*), the second step added primary production (*dynamic1*), the third step added SST, SBT, and SSS (*dynamic2*) and the last step represents the full dynamic model that included red tides (*dynamic3*) (Figure S1).

For each of the four steps in the stability process, we checked response functions and vulnerability parameters of unstable FGs (Figure S1). Several FGs were first identified as unstable during this procedure such as greater amberjack (*Seriola dumerili*), reef invertivores, and gray triggerfish (*Balistes capriscus*). When FGs declined and crashed, we visually explored the spatial overlap (*i.e.* computed foraging capacity) of these FGs with their primary prey items because a mismatch between predators and their prey often leads to FG collapse. If so, environmental response functions were inspected and modified considering alternative data and expert knowledge to improve overlap and thus facilitate predator-prey interaction. For FGs that were still crashing or increasing exponentially, vulnerabilities of their main prey items and/or their major predators were investigated and high vulnerabilities were replaced with lower values that limit how high predation mortality can go (relative to baseline natural mortality rates) and reduces biomass gains by the predator ^1^, thereby increasing the stability of such FGs. We capped vulnerabilities so predation mortality would not exceed a percentage of natural mortality for the prey and calculated the Euclidean distance and root-mean squared error (RMSE) from Ecosim and Ecospace biomass trends for each FG (Figure S2). RMSE was calculated as:

| $RMSE=\sqrt{\frac{\sum{(ŷ_{t}-y_{t})}^{2}}{n}}$ |  |
| --- | --- |

where *ŷ* is the predicted value, *y* is the observed value, *n* is the number of observations and t is the annual time step. After adjusting response functions and vulnerability parameters, we validated Ecospace predictions by comparing the simulated biomass trajectory with Ecosim predicted biomass trends for all living FGs after stability was achieved. For such FGs, the RMSE and the Euclidean distance were calculated between Ecosim and Ecospace biomass (Figure S3). Once the model including all environmental variables was stable, we also checked the RMSE and the Euclidean distance from Ecosim and Ecospace biomass and catch trends while readjusting vulnerabilities with capped vulnerabilities.

After achieving a stable full dynamic model, predicted long-term Ecospace biomass maps were validated with the predicted probability of occurrence maps from GAMs for available FGs. The spatial comparison may highlight FGs that call for improved Ecospace parametrization and/or probability of occurrence maps. Long-term Ecospace biomass maps were obtained by averaging predicted maps of all monthly steps. Schoener’s D and Warren’s I similarity index, and Spearman rank correlation were used for comparing each pair of maps for available FGs (Figure S4). Schoener’s D and Warren’s I similarity index were computed by using the “*ENMTools*” [R] package ^2^.

In the Ecospace calibration process, dispersal and vulnerability values were explored ranging from the base value to the maximum or minimum depending on the previous sensitivity results, and we calculated total pbias as the sum across FGs of absolute weighted pbias values (1,100 scenarios). predictions pbias was chosen over the LL for model selection because pbias predictions resulted in greater improvements overall as it tended to move Ecospace predictions closer to Ecosim. At each iteration of the tuning procedure, we first tested parameter sensitivity by calculating two test values for each dispersal rate and prey-predator vulnerability parameter (914 parameters, 1,830 scenarios). Test values were calculated by adding or subtracting its 0.3 coefficient of variation as follows:

| $\boldsymbol{test}_{\boldsymbol{value}}\boldsymbol{=}\boldsymbol{base}_{\boldsymbol{value}}\boldsymbol{\pm}\boldsymbol{1}\boldsymbol{.}\boldsymbol{96}\boldsymbol{*}\boldsymbol{(}\boldsymbol{0}\boldsymbol{.}\boldsymbol{3}\boldsymbol{*}\boldsymbol{base}_{\boldsymbol{value}}\boldsymbol{)}$ |  |
| --- | --- |

FG pbias values were weighted considering the focus of this study, and so high weights were assigned to gag grouper, red grouper, and managed species FGs (see Supplementary Table S1). Regarding dispersal rates, tested values were bounded between 3 and 300 km·year^-1^. For vulnerabilities, if the first test value was higher than the base value, we then tested additional values from 1.5 times the base value to 10,000. If the vulnerability test value was lower than the base value, we tested values ranging from 1.01 to 0.9 times the base value. We selected the prey-predator vulnerability and dispersal rate values with the lowest total pbias. We then compared the total pbias of the base model run, a model including the prey-predator vulnerability and dispersal rate with the lowest pbias values, and a model including only the prey-predator vulnerability with the lowest pbias value. Among these three configurations, we selected the model with the lowest pbias that represented the new base model for the next iteration, with previously selected parameters excluded. The iterative process stopped when there was no improvement to the pbias which occurred after 35 iterations. Overall, the fitting procedure included more than 80,000 Ecospace runs. This was achieved by using a multicore computer (48 cores) in parallel that allowed me to run multiple Ecospace runs simultaneously and faster.

## Supplementary Figures


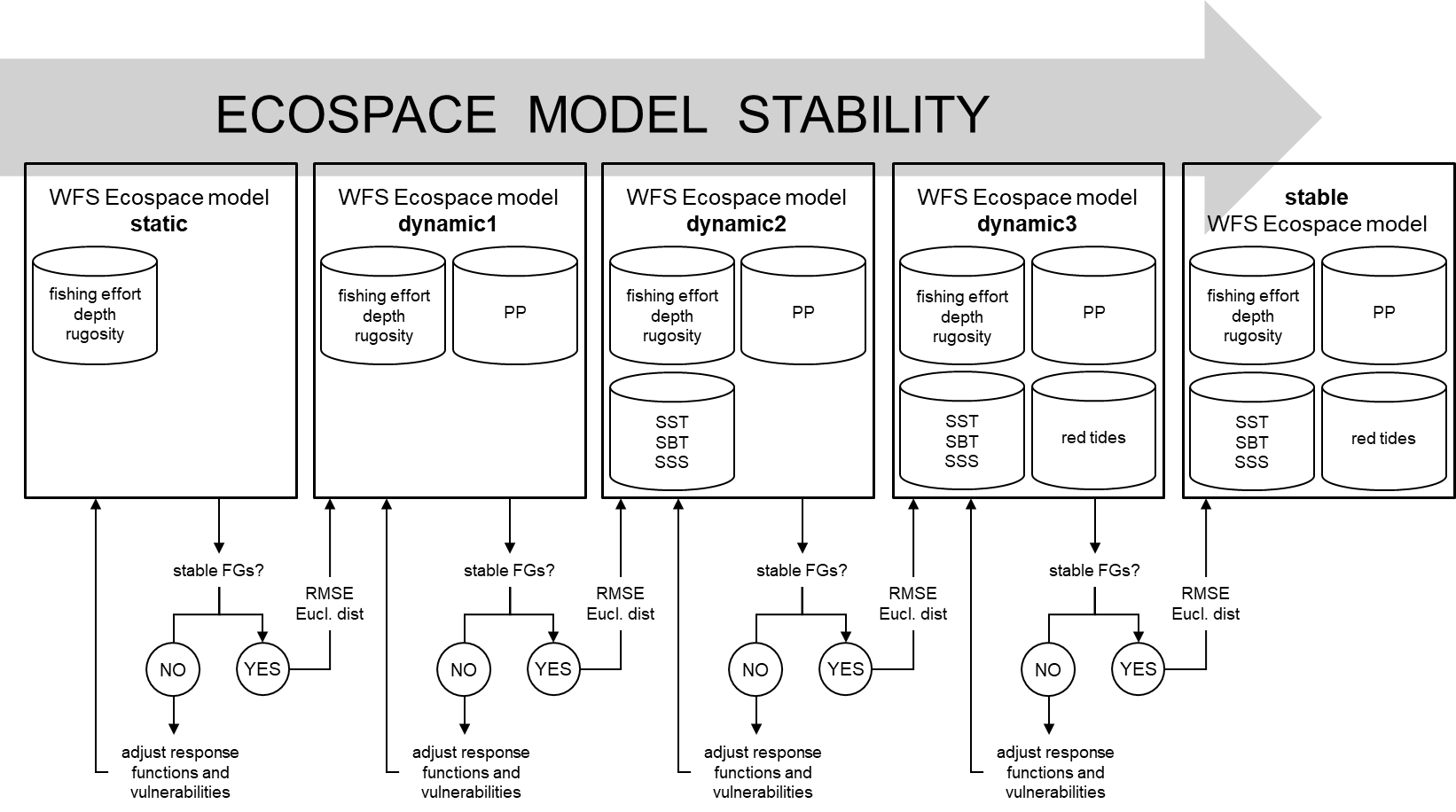


Figure S1. Flowchart of the stepwise stability process for spatiotemporal ecosystem models by sequentially adding model complexity in terms of the incorporation of environmental variables that drive the foraging capacity of functional groups, going from a static to fully spatially dynamic Ecospace model (dynamic3).


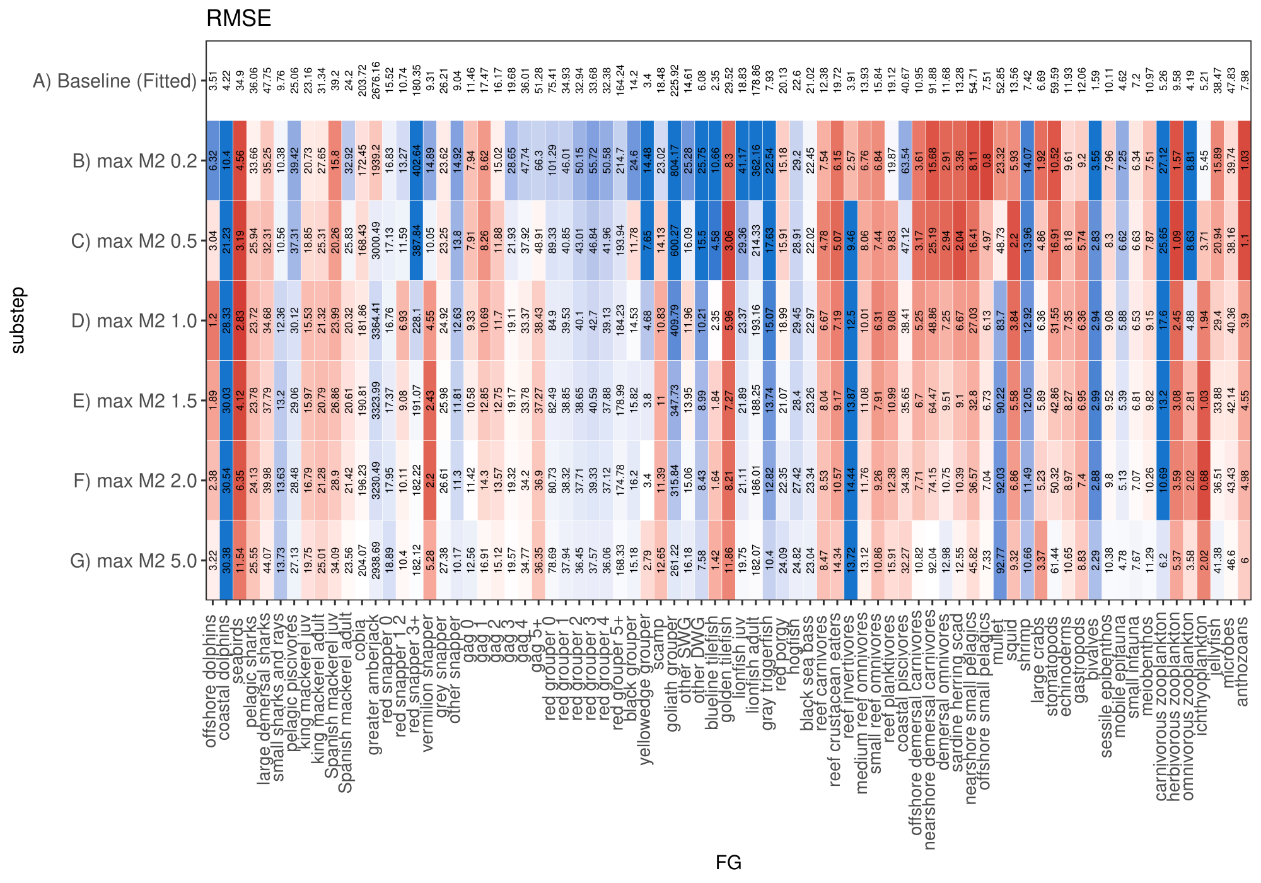

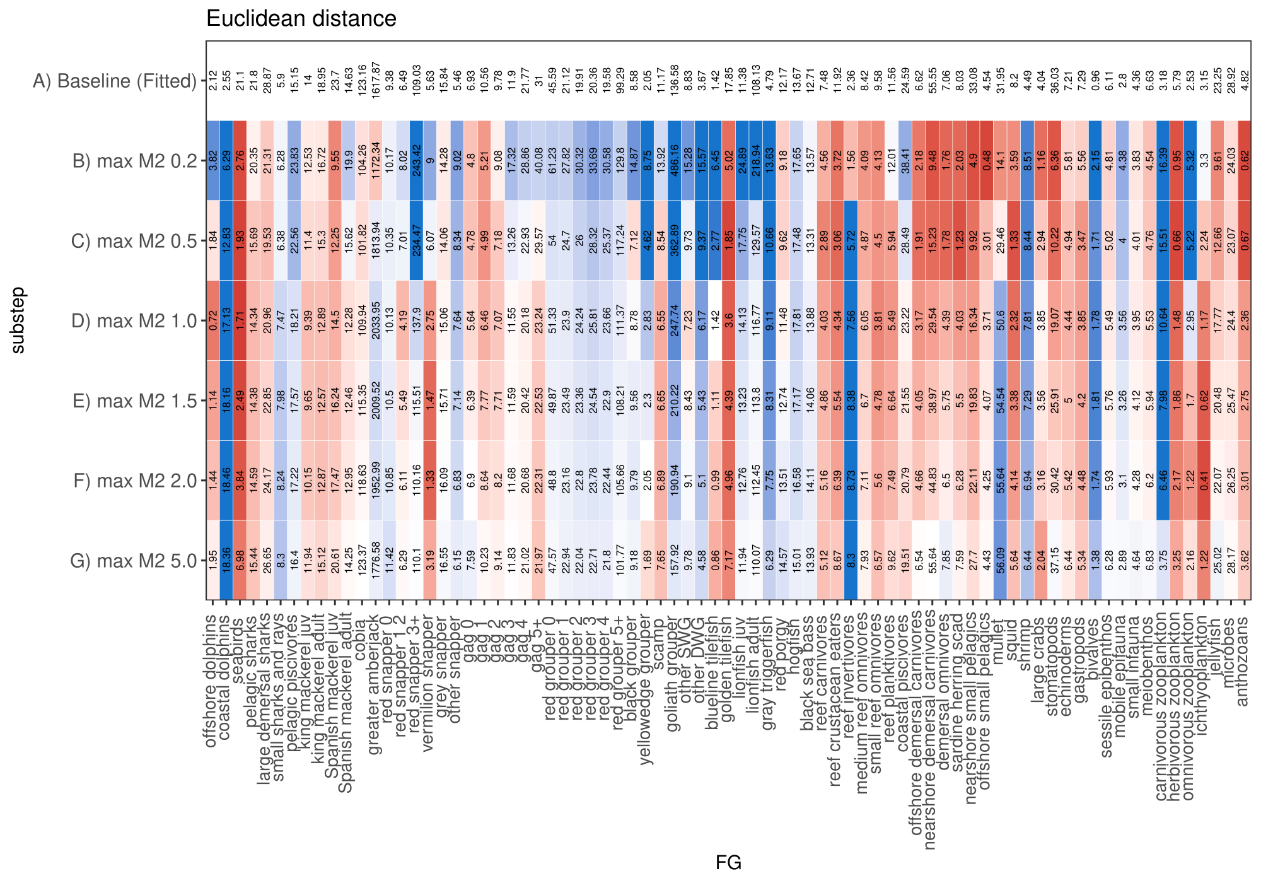


Figure S2. Root-mean square error (RMSE) and Euclidean distance values between Ecosim and Ecospace predictions for each substep of the capped vulnerability investigation analysis. Color represents lower (red) or higher (blue) RMSE or Euclidean distance compared to the baseline scenario.


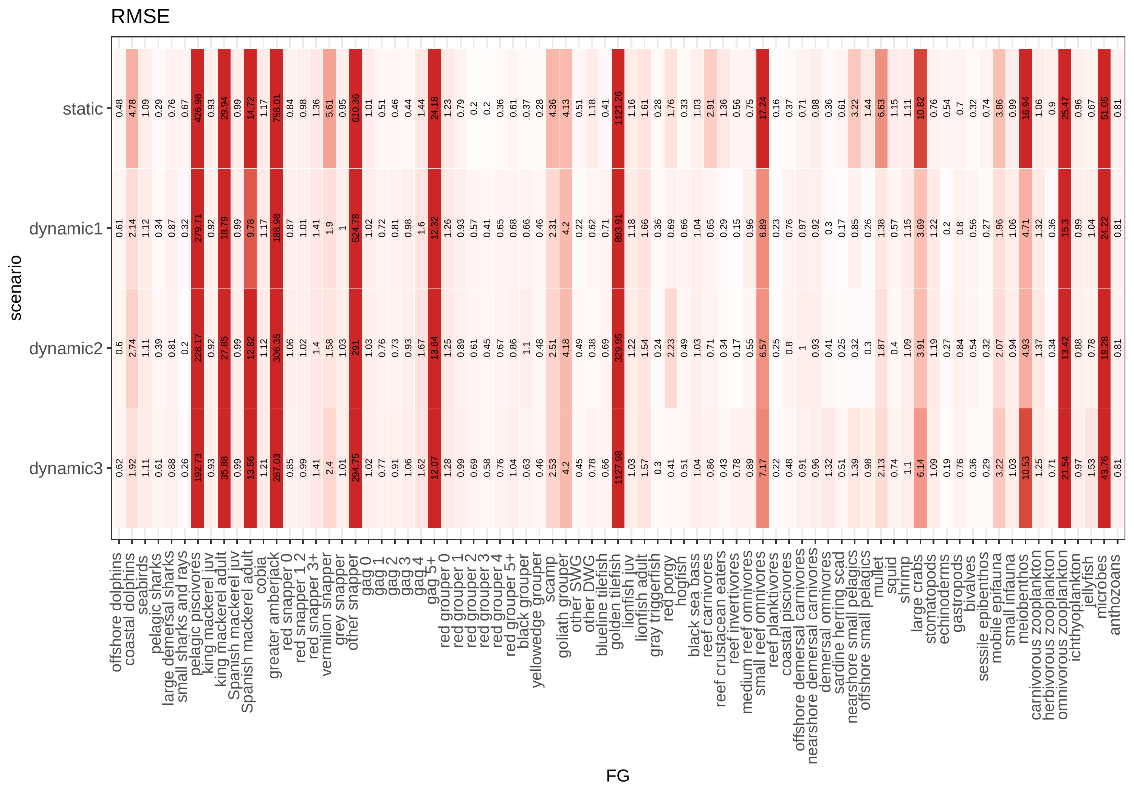

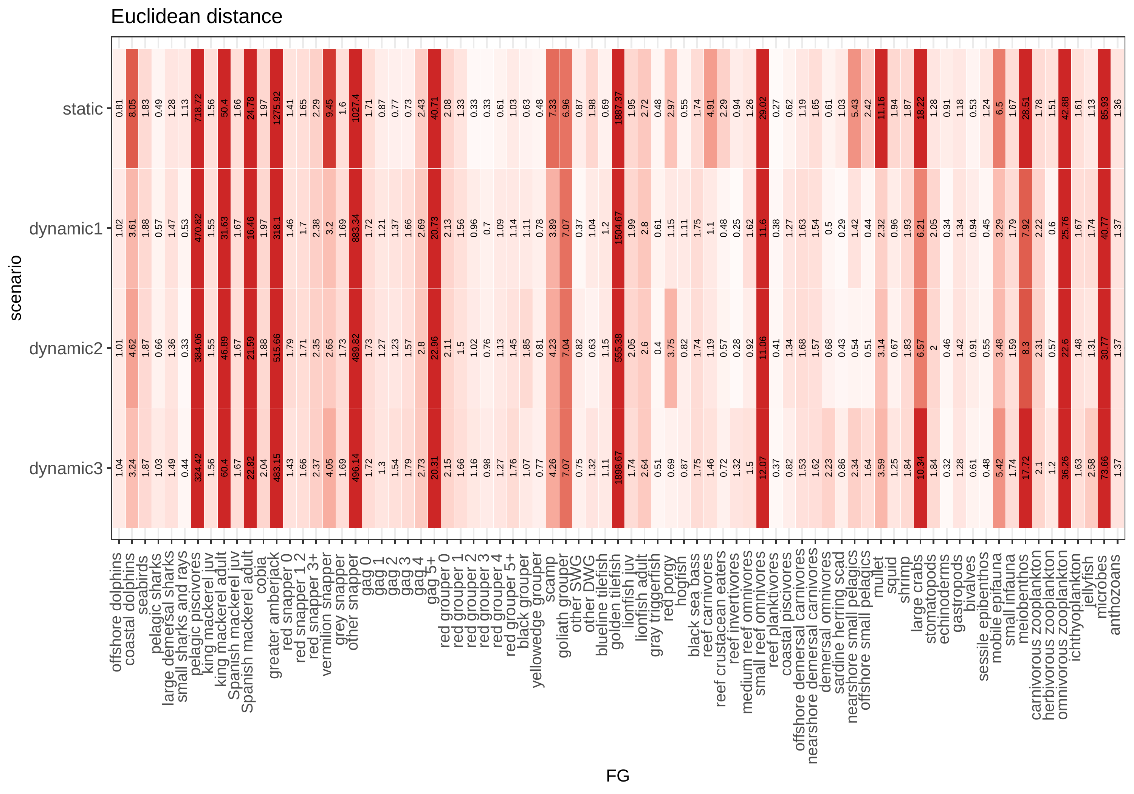


Figure S3. Root-mean square error (RMSE) and Euclidean distance values between Ecosim and Ecospace predictions for each scenario of the stability stepwise Ecospace process. Each scenario identifies the following structure: static fishing effort, depth, and rugosity; dynamic1 fishing effort depth, rugosity, and primary production; dynamic2 fishing effort, depth, rugosity, primary production, SST, SBT, and SSS; and dynamic3 the full dynamic model that included red tides. Red color intensity represents the value of RMSE and Euclidean distance.


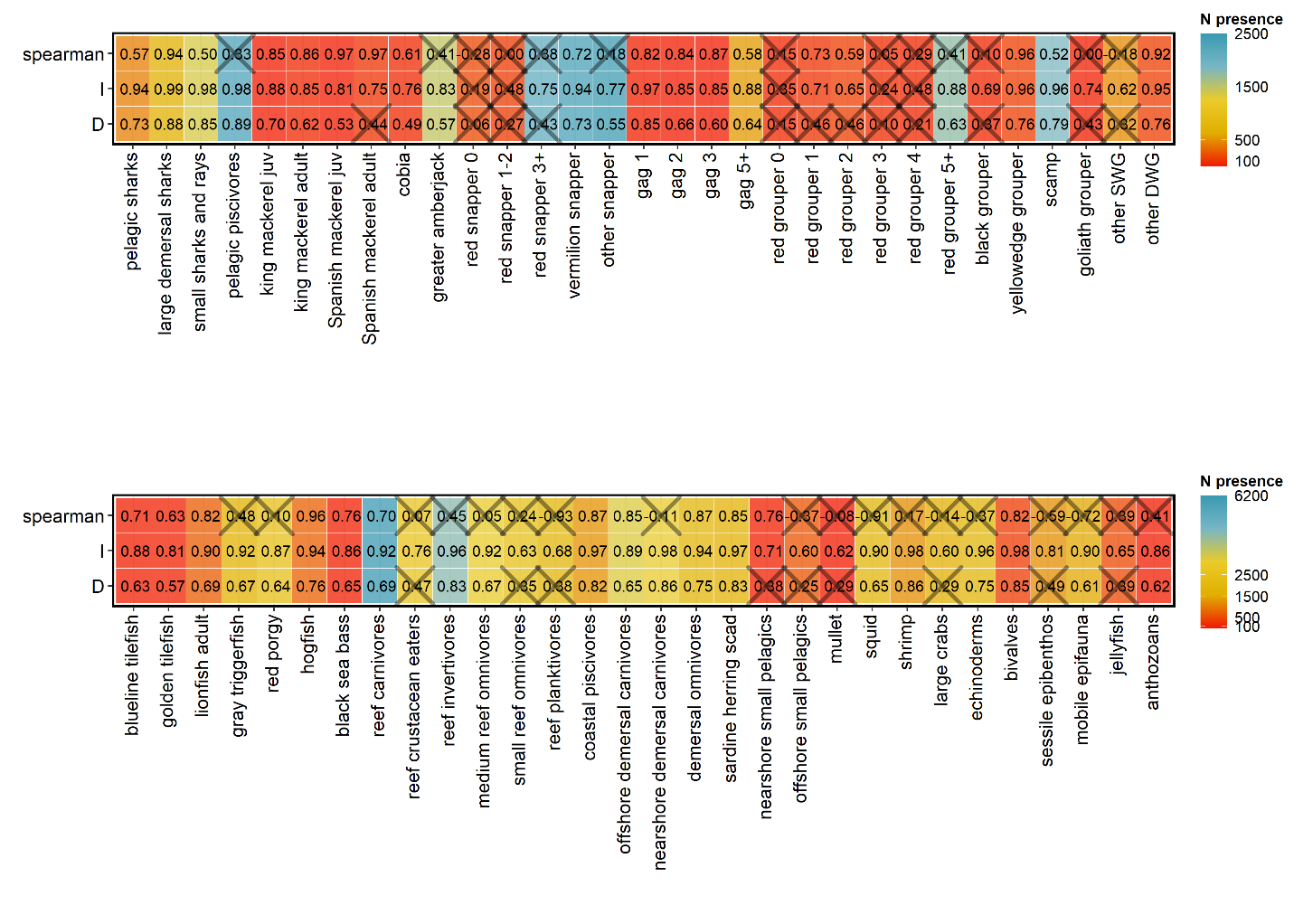


Figure S4. Spearman rank correlation coefficients and Warren’s I and Schoener’s D similarity index values of each available functional group for the Ecospace validation procedure. Colors indicate the number of presence data to calculate environmental response functions and FG distributions. ‘X’ symbol represents a low level of correlation between rasters (I<0.5; D<0.5; Spearman correlation coefficient<0.5).


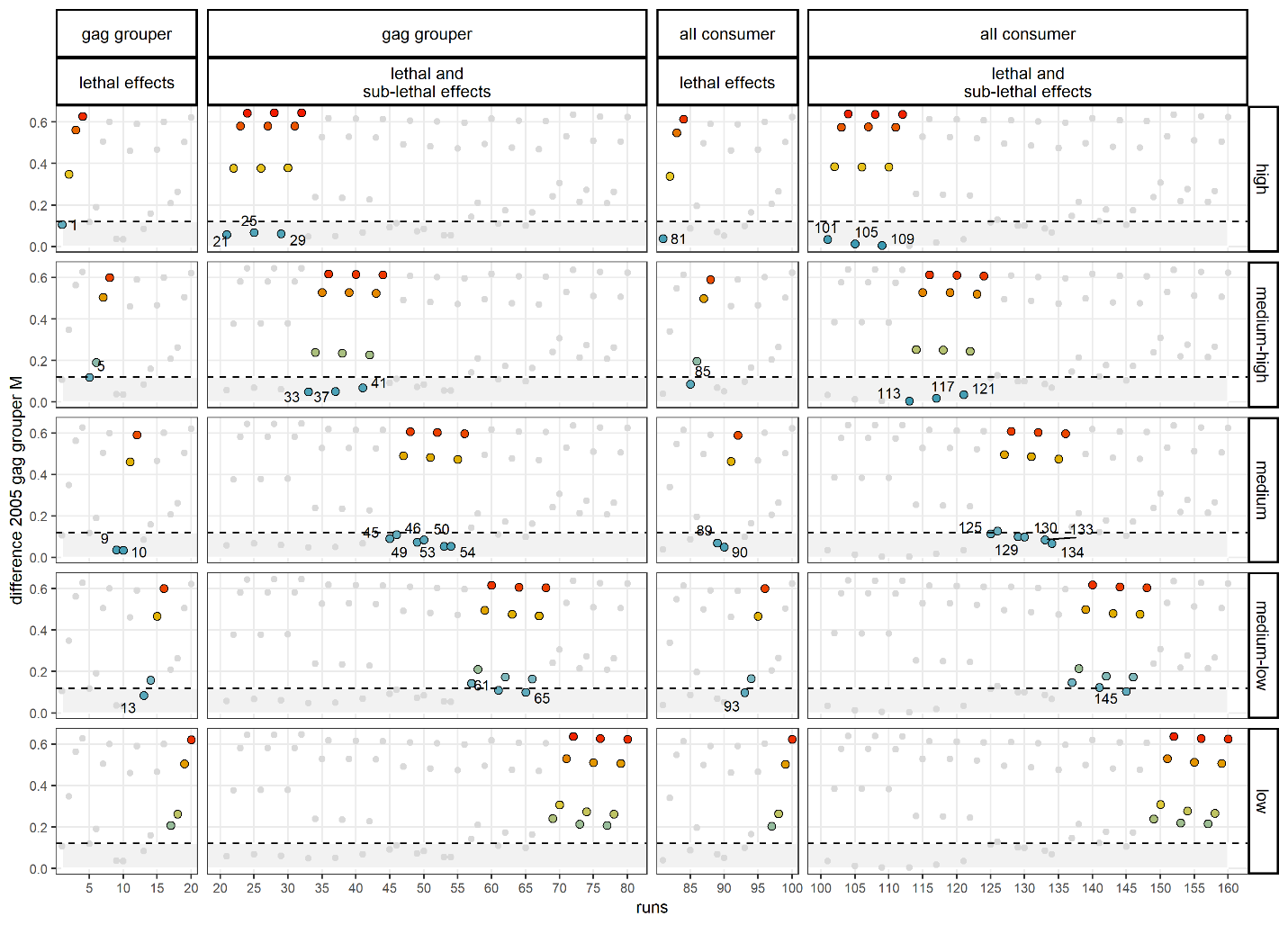


Figure S5. The absolute difference in gag mortality in 2005 estimated in SEDAR 72 for each of the 160 red tide scenarios classified by sensitivity to red tides, functional groups in which red tide effects were applied (gag - direct red tide effects and all consumer – indirect food-web red tide effects), and red tide effects applied (lethal and sublethal effects. Color intensities represent the magnitude of the estimate (red: high; blue: low). Numbers identify feasible scenarios (for detailed information on red tide scenarios see Table S3).


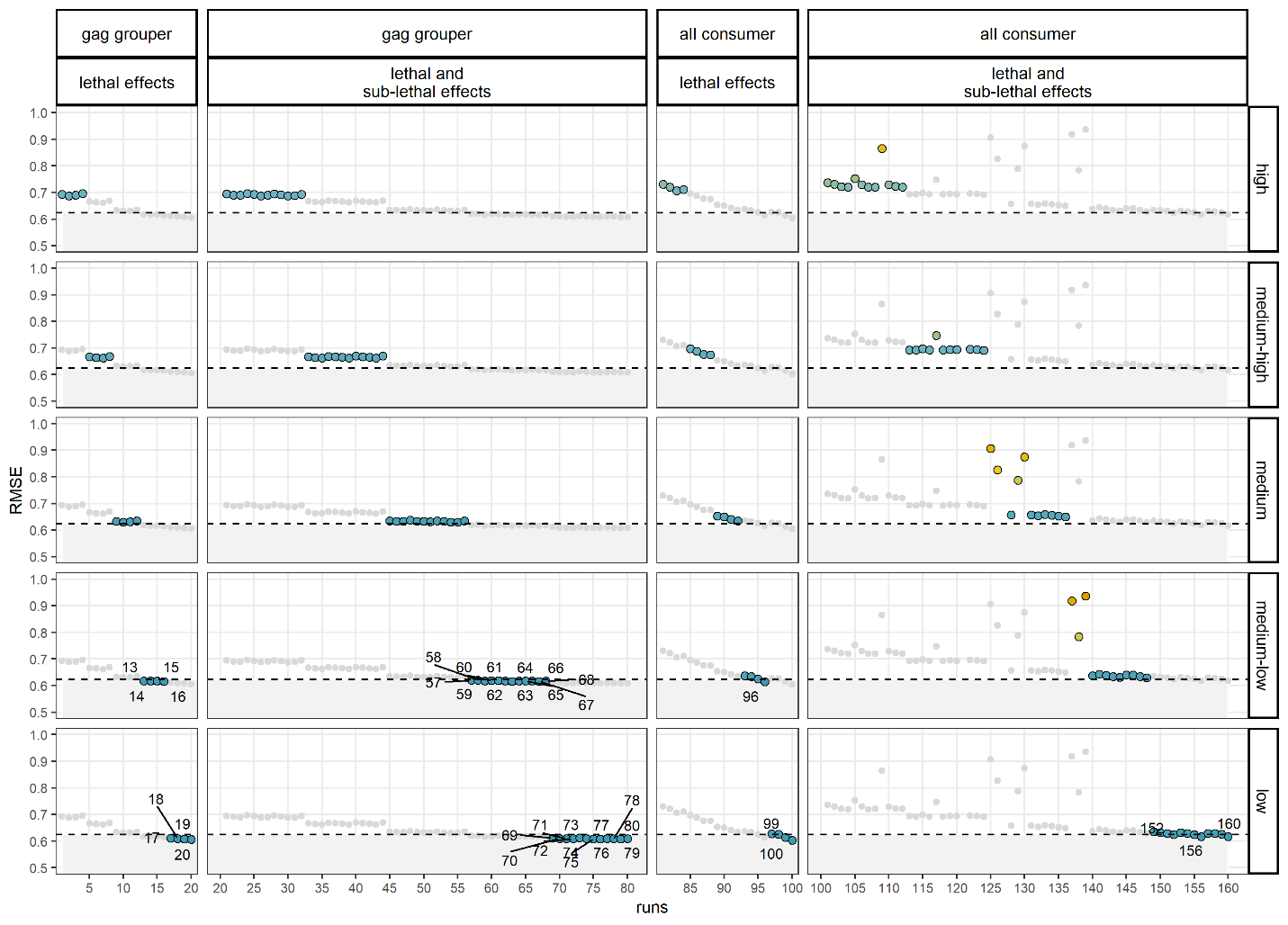


Figure S6. Root-mean squared error (RMSE) for each of the 160 red tide scenarios classified by sensitivity to red tides, functional groups in which red tide effects were applied (gag - direct red tide effects and all consumer – indirect food-web red tide effects), and red tide effects applied (lethal and sublethal effects). Color intensities represent the magnitude of the estimate (red: high; blue: low). Numbers identify feasible scenarios (for detailed information on red tide scenarios see Table S3).

## Supplementary References

1. Chagaris, D. *et al.* An ecosystem-based approach to evaluating impacts and management of invasive lionfish. *Fisheries* **42**, 421–431 (2017).

2. Warren, D. L. *et al.* ENMTools 1.0: an R package for comparative ecological biogeography. *Ecography* (2021).
